# Extreme Weather Magnifies the Effects of Forest Structure on Wildfire, Driving Increased Severity in Industrial Forests

**DOI:** 10.1111/gcb.70400

**Published:** 2025-08-20

**Authors:** Jacob I. Levine, Brandon M. Collins, Michelle Coppoletta, Scott L. Stephens

**Affiliations:** ^1^ Wilkes Center for Climate Science and Policy University of Utah Salt Lake City Utah USA; ^2^ School of Biological Sciences University of Utah Salt Lake City Utah USA; ^3^ USDA Forest Service Pacific Southwest Region Vallejo California USA; ^4^ Department of Environmental Science, Policy, and Management University of California Berkeley Berkeley California USA; ^5^ USDA Forest Service Sierra Cascade Province Ecology Program Quincy California USA

**Keywords:** climate change, extreme weather, fire severity, forest management, forest structure, industrial forests, ladder fuels, ownership, plantations

## Abstract

Despite widespread concern over increases in wildfire severity, the mechanisms underlying this trend remain unclear, hampering our ability to mitigate the severity of future fires. There is substantial uncertainty regarding the relative roles of extreme weather conditions, which are exacerbated by climate change, and forest management, in particular differences between private industrial timber companies and public land agencies. To investigate the effects of extreme weather and forest management on fire severity, we used light detection and ranging (LiDAR) data to characterize pre‐fire forest structure across five large wildfires which burned 460,000 ha in the northern Sierra Nevada, California, USA. We found that the odds of high severity fire occurrence in these fires were 1.45 times higher on private industrial land than in publicly owned forests, an effect equivalent to a three standard deviation decrease in fuel moisture. Next, we quantified the relationships between key forest structure metrics and the probability of high severity fire, as well as how these relationships were modified by extreme weather. We found that dense, spatially homogeneous forests with high ladder fuels were more likely to burn at high severity. Extreme weather magnified the effect of density, suggesting that treatments which remove overstory trees are especially important in extreme conditions. Forests managed by private industry were more likely to be dense, spatially homogeneous, and contain high ladder fuel loads than publicly owned forests, offering a potential explanation for the increase in high‐severity fire occurrence on private industrial land. Overall, these results illustrate the need for comprehensive forest management to mitigate fire severity in a warmer future.

## Graphical abstract

Wildfires are consistently more severe in private industrial forests than public ones. In this paper, we identify the drivers of this pattern. Namely, we show that fire severity is elevated in dense, spatially homogenous forests with high ladder fuels—characteristics more common on private industrial land than adjacent public land. Moreover, we find that the effects of forest structure are magnified in extreme weather conditions, suggesting that efforts to reduce density and ladder fuels, and increase heterogeneity, will only become more critical as the Earth warms.

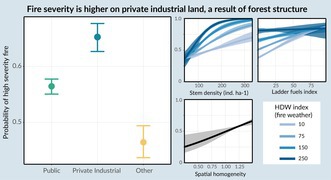

## 1 Introduction

Widespread increases in the size, frequency, and severity of wildfires over the past several decades threaten ecological and social systems both in the western United States and globally (Abatzoglou and Williams 2016; Burke et al. 2021; Parks and Abatzoglou 2020; Steel et al. 2018). The proliferation of high‐severity fire, in which more than 95% of overstory trees are killed (Parks et al. 2016; Parks and Abatzoglou 2020), is particularly concerning because many forest types do not regenerate following complete or near‐complete overstory mortality (Coop et al. 2020; Davis et al. 2019). As a result, extensive high‐severity fire can spur conversion to non‐forest ecosystem types such as shrublands, leading to loss of wildlife habitat (Jones et al. 2016) and reduced carbon sequestration potential (Coop et al. 2020). Even in intensively managed forests where active reforestation is more likely, high‐severity fire and associated fire behavior pose a significant hazard to timber resources, water quality, forest carbon, and human health and safety (Bousfield et al. 2023; Reid et al. 2016).

There is significant uncertainty regarding the causes of recent increases in high‐severity fire incidence. Though forest management and extreme weather are known to affect severity, the relative importance of these two drivers is hotly debated in both scientific journals (Bradley et al. 2016; Miller et al. 2012; Odion et al. 2004; Peery et al. 2019) and the public sphere (Hudson 2011). Moreover, while there is widespread agreement that climate change will raise high‐severity fire risk by fostering more extreme weather conditions (Abatzoglou and Williams 2016; Parks et al. 2016), there is less agreement over which forest management practices most strongly affect severity, or whether the effects of forest management are more or less important under extreme weather conditions (Bradley et al. 2016; Lydersen et al. 2017; Lyons‐Tinsley and Peterson 2012).

Much of the debate surrounding forest management's role in fostering or mitigating high‐severity fire centers on differences between private industrial timber companies and public land management agencies such as the United States Forest Service and National Park Service (Schwartz et al. 2020). Forests managed by private industry are typically associated with more intensive plantation forestry (Koontz et al. 2020; Zald and Dunn 2018), which is employed to maximize sustainable timber production (Sedjo 1999). However, the resulting even‐aged structure tends to be relatively homogeneous with greater overall fuel continuity (Zald and Dunn 2018), characteristics that contribute to more extreme fire behavior and higher fire severity (Francis et al. 2023). In contrast, public land management agencies must balance multiple objectives in addition to timber production, such as recreation, rangeland management, and wildlife conservation. Moreover, these agencies are subject to greater public scrutiny and litigation, generally resulting in less intensive forest management (Collins et al. 2017; Mooney and Zavaleta 2016). Because of this and a long‐established policy of fire removal from fire‐adapted forests, publicly managed forestland is commonly overly dense and fuel‐laden, which also contributes to extreme fire behavior (Starrs et al. 2018).

Recent research has begun to shed light on the relationship between management and fire severity. For example, an analysis of 154 wildfires across the state of California found that the odds of burning at high severity were 1.8 times higher in private industrial forests than on public land (Levine et al. 2022), corroborating the findings of a previous single‐fire study (Zald and Dunn 2018). However, we have little understanding of the mechanisms driving increased severity on private industrial land, hampering our ability to effectively mitigate fire severity in the future. Moreover, the fires in these studies occurred in prior decades, when the effects of climate change were markedly less pronounced (Flannigan et al. 2009). It remains unclear whether, in the new era of “mega‐fires,” these results will hold, or if the effects of extreme weather overwhelm the influence of forest management.

**FIGURE 1:**
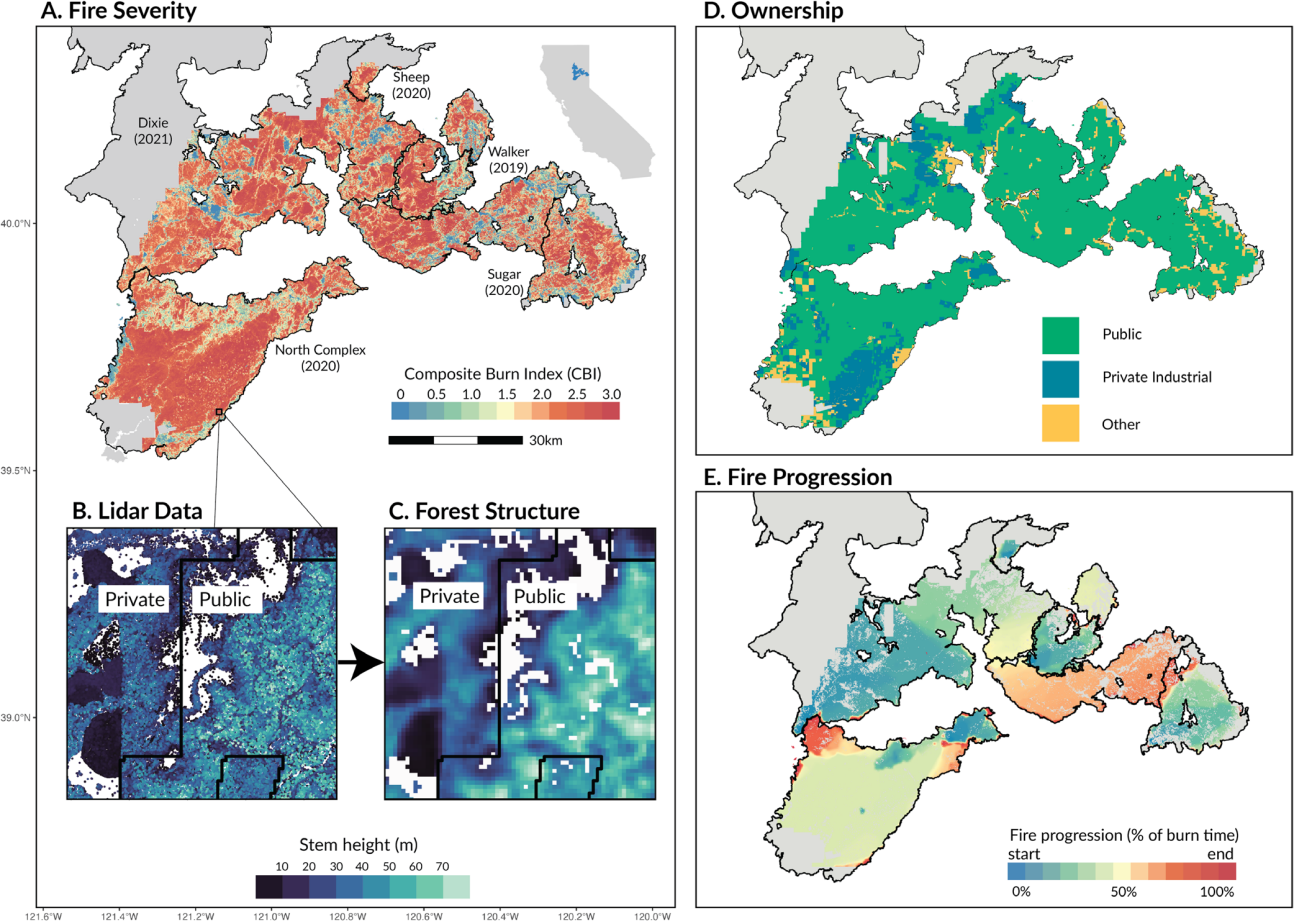
Study overview. (A) shows fire severity in the five study fires as quantified by the composite burn index (CBI). Areas in gray indicate regions which were within a fire perimeter, but which were excluded from the study because they fell outside the extent of our LiDAR dataset [collection perimeter]. The inset map in the upper‐right corner of (A) shows the location of these five fires within the US state of California. (B) shows a map of individual trees within a small patch of the area displayed in Panel A, illustrating the forest structure data derived from the aerial LiDAR data. Colors in Panel C correspond to the height of individual trees. Black lines indicate property ownership boundaries between private industry (left) and the United States Forest Service (right). The difference in forest structure between land owned by private industry and the public is evident. Particularly, the private industrial land contains several homogeneous patches of trees of similar height, characteristic of plantation management, whereas the right side exhibits high heterogeneity in both tree height and the spatial distribution of stems and forest gaps. (C) demonstrates how the individual tree data was processed into forest structure metrics, where each 30 × 30 m pixel shows the average tree height in a larger 90 m by 90 m window centered on that pixel. (D) displays land ownership patterns over the full study area. (E) shows the estimated progression of each fire in the study area. Fire progressions were estimated using visible infrared imaging radiometer suite (VIIRS) data from NASA, and were integral for quantifying weather at a high spatial and temporal resolution. In the plot, progressions are normalized by the total length of the fire, such that the color corresponds to the percent of the total fire length at which a pixel burned. Map lines delineate study areas and do not necessarily depict accepted national boundaries.

The northern Sierra Nevada, California, USA, embodies many of the broader trends in wildfire occurrence and severity and provides a unique opportunity to investigate the drivers of high severity fire. The area contains a patchwork of private industrial and public ownership typical of the western USA (Figure 1), and in just 3 years between 2019 and 2021, 70% (4595 km^2^) of the area (as defined by the boundaries of the Plumas National Forest, California, and adjacent private land) burned in five large fires, 39% (2527 km^2^) of it at high severity (Figure 1). At the center of this unprecedented period was 2021's Dixie Fire, the largest single fire in California's recorded history, burning just under 400,000 ha of public and private lands (Figure 1).

In 2018, 1 year before the first of these fires started, the US Forest Service collaborated with the United States Geologic Survey (USGS) and the National Aeronautics and Space Administration (NASA) to collect airborne light detection and ranging (LiDAR) data across the Plumas National Forest and adjacent private lands. These LiDAR data were used to map locations of individual trees > 4‐m in height. The result is a detailed pre‐fire characterization of forest structure, which allowed us to investigate not only differences in high‐severity fire incidence between private industrial and public lands, but also the mechanisms underlying these differences.

Here we leverage this high‐fidelity dataset to investigate three questions: (1) Is the incidence of high‐severity effects in these recent wildfires greater on private industrial or public land? (2) Which forest structure, climate, and spatial characteristics are associated with high‐severity fire, and how are these relationships modified by extreme weather conditions? and (3) Can differences in fire severity across ownership types be explained by the forest structures generated by private industrial versus public management?

## 2 Materials and Methods

### 2.1 Data and Study Design

#### 2.1.1 Study Area

To answer these questions, we analyzed patterns of burn severity in five wildfires that burned a collective 459,554 ha within the footprint of the Plumas National Forest, northern California, USA: the Dixie, North Complex, Sugar, Sheep, and Walker Fires (Figure 1; Table S1). Though these fires also burned an additional 100,000+ hectares in neighboring areas, the study region was restricted to the Plumas National Forest and adjacent private land due to the availability of LiDAR data. All fires began on public land. Three of the fires were the result of lightning, while the Dixie Fire was started by a damaged electrical distribution line, and the source of the Walker Fire is unknown.

Forests in this region are primarily comprised of yellow pine and mixed‐conifer forest types, dominated by ponderosa pine ( *Pinus ponderosa* ), sugar pine ( *Pinus lambertiana* ), Douglas‐fir ( *Pseudotsuga menziesii* ), incense‐cedar ( *Calocedrus decurrens* ), white fir ( *Abies concolor* ), black oak (*Quercus kellogii*) and Jeffrey pine ( *Pinus jeffreyi* ), with smaller portions of red fir ( *Abies magnifica* ), quaking aspen ( *Populus tremuloides* ), and lodgepole pine ( *Pinus contorta var. murrayana* ). Most of these species are adapted to frequent, low‐severity fire regimes, with a historical average fire return interval in this region of 6–20 years (Coppoletta et al. 2024; Moody et al. 2006). The study area spans both the east and west slopes of the Sierra Nevada, including elevations ranging from 254 m in the western foothills to 2540 m at the Sierra Nevada crest.

A large majority of the study area (81.6%; after removing non‐forested pixels) was managed by public land agencies, primarily the United States Forest Service, whereas 11.7% was owned by private industrial timber companies, and the remaining 6.6% by non‐industrial private entities (we refer to this third category as “other” throughout the manuscript; Figure 1B). Land ownership was determined using the Fire and Resource Assessment Program's (FRAP) ownership database (https://frap.fire.ca.gov/mapping/gis‐data) and a map of industrially managed timberland in California assembled using property records (T Moody personal communication; Figure 1B). Finally, we restricted our analysis to include only those 30 × 30 m pixels with > 10% cover of trees over 4 m tall. We did this to avoid analyzing non‐forested pixels. The final dataset contained 3,315,523 pixels, or just under 300,000 ha.

#### 2.1.2 Estimating Fire Severity

Fire severity was quantified using the bias‐corrected composite burn index (CBI) at a 30 m resolution, which we estimated using a random forest model developed by Parks et al. (2019). Specifically, this model uses Landsat imagery taken immediately before and 1 year after each fire, as well as information on local geography and climate, to quantify CBI based on established empirical relationships. To clarify that CBI is estimated from remote sensing data rather than on‐the‐ground measurements, we refer to it as “satellite‐estimated CBI” throughout. After estimating CBI, we categorized each pixel as burning at “high severity” or “low‐moderate severity” using an empirically derived CBI threshold (CBI > 2.25; Miller and Thode 2007).

Though satellite‐estimated CBI is not a direct measure of fire severity, which requires field measurements of tree mortality, studies have demonstrated reliable correlations between satellite‐estimated CBI and overstory mortality (Lydersen et al. 2016). This is particularly true for the distinction between “low‐moderate severity” and “high severity,” which regularly corresponds to over 95% live basal area mortality in empirical studies (Lydersen et al. 2016; Miller and Thode 2007; Parks et al. 2019). We chose to quantify severity as a binary outcome because of the distinct ecological and economic consequences of high‐severity fire, which can spur conversion to non‐forest ecosystem types (Coop et al. 2020; Davis et al. 2019).

#### 2.1.3 Quantifying Forest Structure

We used datasets derived from two airborne LiDAR acquisitions that covered the entirety of our study area and were collected by the USGS and NASA in the summer and fall of 2018–2019. The Forest Service Mapping and Remote Sensing Team used these LiDAR data to identify the approximate locations and tree crown area of individual trees > 4‐m in height. Individual trees were delineated using the TreeSeg tool in the FUSION software package version 4.20 (McGaughey 2007). TreeSeg applies a watershed transformation algorithm to a high‐resolution canopy height model (CHM) to identify the highest point of each vegetation feature. These high points are then interpreted as approximate locations of individual trees, frequently referred to as “tree approximate objects” (Jeronimo et al. 2018). For simplicity, we refer to these objects as “trees” or “stems” throughout this article. Individual tree crowns were estimated using an algorithm in TreeSeg that starts at the high point and identifies the edge (i.e., where canopy height drops below a threshold height or starts to increase) in 18 evenly spaced radial profiles (McGaughey 2007). CHMs were created using a 0.75‐m cell size that was smoothed with a 3 × 3 lowpass filter. FUSION‐generated estimates of first return density ranged from 13.2 to 15.9 returns/m^2^ across the project area.

Using the data processed with TreeSeg, we further derived five forest structure metrics: mean stem density per hectare (trees > 4 m tall), mean stem height, mean gap area, spatial homogeneity, and a “ladder fuels index.” These metrics were chosen because (1) they are thought to play a key role in determining fire behavior and (2) they correspond to perceived differences between public and private industrial management. Each of these metrics, except for the ladder fuels index, was quantified at two spatial scales: a 90 m by 90 m box and a 390 m by 390 m box, both centered on each 30 m by 30 m pixel. The 90 m by 90 m (~1 ha) scale was intended to capture the effects of the immediate neighborhood of a pixel on fire behavior, whereas the 390 m by 390 m (~15 ha) scale was intended to capture dynamics at the stand scale (15 ha is slightly larger than the maximum allowed clear‐cut in California).

To calculate mean stem density, we simply summed the total number of individual tree stems within each bounding box (90 m by 90 m or 390 m by 390 m) and divided by the area. LiDAR often fails to identify small trees, especially under dense canopy layers, meaning stem density is likely underestimated in this study. Mean tree height was calculated as the average of all individual tree stems within the bounding box. To determine average gap area, we used tree canopy polygons to first delineate all gaps within a bounding box (both neighborhood and stand scale)—a gap being defined as any contiguous area of forest not occupied by a tree's canopy. Gaps were only quantified within a given bounding box, meaning if a gap extended past the boundary of the 90 m by 90 m or 390 m by 390 m area, the portions falling outside the box were excluded. Finally, we calculated the size of each gap within the box and took the average.

The spatial homogeneity metric is intended to describe the degree of clustering versus regularity in the location of individual tree stems. To place pixels along this continuum, we divided the average nearest neighbor distance of trees within a bounding box by the expected nearest neighbor distance had each tree been placed at random according to a Poisson point process. The result is the following equation, adapted from (Clark and Evans 1954):

(1) $$h=\frac{\frac{1}{N}\sum_{i\neq j}^{N}\min\left[u_{i,j}\right]}{\frac{1}{2}\sqrt{\frac{A}{N}}}$$

where N is the number of stems in the bounding box, $u_{i,j}$ is the distance in meters between stem i and stem j, and A is the area of the bounding box in square meters.

Ladder fuels allow fire to spread from the forest floor into the canopy and are thus critical drivers of fire severity. These can be small trees, low tree branches, shrubs, and/or downed wood that generate a continuous vertical fuel profile between the forest floor and canopy. To characterize the relative amount of ladder fuels, we developed a metric that captures both the vertical continuity and total amount of vegetation in a pixel. To derive this metric, we used data on vegetation cover in four height bands: 2–8, 8–16, 16–32, and 32 m+, which have historically been used to characterize similar forest types in analyses of ladder fuels, fire impacts, and critical habitat delineation (Kane et al. 2014, 2019; Kramer et al. 2016; North et al. 2017). These cover data were processed from the raw point cloud data by the Forest Service Mapping and Remote Sensing Team using the “Cover” function in the FUSION software package. Then, the continuity in cover across these four height bands was quantified by adapting Simpson's evenness index (Morris et al. 2014), which is equal to one when all four bands have the same cover, and less than one when there is heterogeneity in cover across bands. Multiplying this evenness index by the average cover across all four bands gives the ladder fuels index:

(2) $$l=\mu_{c}{\left[q\sum_{k=1}^{q}{\left(\frac{c_{k}}{\sum_{j=1}^{q}c_{j}}\right)}^{2}\right]}^{-1}$$

where $\mu_{c}$ is the mean cover across the bands, q is the tallest band with cover greater than zero, and $c_{j}$ is the canopy cover of band j. This metric is similar to several prior approaches developed to quantify ladder fuels from LiDAR imagery (Hakkenberg et al. 2024; Kramer et al. 2016). However, whereas past efforts focus primarily on the total cover in low canopy strata, we integrate both total cover and vertical continuity.

Unlike the other forest structure metrics in our analysis, we calculated the ladder fuels index only at the pixel scale (30 m by 30 m). There were two reasons for this. First, the ladder fuels index is highly collinear with stem density and average gap area, meaning it would be difficult to separate its effect on severity from the effects of these metrics if measured on the same spatial scale. Second, ladder fuels most strongly affect the mortality of trees immediately above them. While there are certainly secondary effects at larger scales, these are both less pronounced and likely captured by the other metrics in our analysis.

#### 2.1.4 Weather, Climate, and Topographic Indices

To characterize the weather experienced by each 30 m by 30 m pixel at the time it burned, we used the average of nearby remote automated weather stations (RAWS), weighted by distance and elevation difference (Figure S1). We used two weather indices known to strongly influence fire behavior: the hot‐dry‐windy index and fuel moisture. The hot‐dry‐windy index is a composite metric calculated by multiplying wind speed and vapor pressure deficit (a function of temperature and relative humidity; Srock et al. 2018). Fuel moisture is simply the percent moisture content of 10‐h woody fuels, measured using an instrument designed to emulate medium‐sized twigs on the forest floor.

A key step in quantifying these metrics was to determine the window in which each pixel burned. Typically, the low temporal and spatial fidelity of weather data hinders analyses of its relationship to fire effects (e.g., Levine et al. 2022). To overcome this limitation, we took advantage of visible infrared imaging radiometer suite (VIIRS) data from NASA. VIIRS measures heat incidence on the ground at regular intervals and can therefore be used to delineate wildfire activity at a 375‐m resolution (Briones‐Herrera et al. 2020; Stephens et al. 2022). Using this data, we developed an algorithm (modified from Briones‐Herrera et al. 2020) to estimate the 8‐h time window in which each pixel in the study area burned. See Appendix 1 for a complete description of the algorithm. Weather indices were then averaged across each time window.

We also took advantage of these temporal data to quantify average fire severity in the period before each pixel burned (henceforth “incoming severity”). Estimating the incoming severity for each pixel allowed us to control for the fire's effects as it approached a given area. Specifically, for each pixel in the study region, we took a spatially weighted average of severity (satellite‐estimated CBI) for a random sample of 1000 pixels that burned in the previous 8‐h window.

All topographic indices (slope, topographic position index, and heat load) were calculated from the US National Elevation Dataset 30 m digital elevation model (Gesch et al. 2002). Slope was calculated using the “terra” package version 1.6‐47 (Hijmans et al. 2022) in R version 4.2.2 (R Core Team 2013). Topographic position index (TPI), which characterizes a pixel's elevation relative to nearby topography (i.e., top of a ridge versus bottom of a canyon) was calculated as the difference between a pixel's elevation and the average elevation of all pixels within a 300 m radius. We chose a 300 m annuli because previous studies have demonstrated a clear association between TPI and fire severity at that scale (Levine et al. 2022; Zald and Dunn 2018). Heat load, which describes incident radiation, was calculated from slope, aspect, and latitude following the methods of McCune and Keon (2002). To account for bioclimatic differences across the study area, we also analyzed data on climatic water deficit from the California Basin Characterization Model (Flint et al. 2013).

### 2.2 Statistical Analyses

#### 2.2.1 Assessing the Impact of Ownership on Fire Severity

To determine whether the incidence of high‐severity effects in our five study fires was greater on private industrial or public land, we fit a binomial generalized linear model to estimate the probability of a 30 m by 30 m pixel burning at high severity as a function of ownership category (private industrial, public, or other). Because these ownerships exhibit substantial differences in both their biophysical characteristics and the weather conditions experienced during the fire, we also controlled for two key weather metrics (hot‐dry‐windy index and fuel moisture), three topographic indices (slope, topographic position index, and heat load), climatic water deficit, incoming severity, and a unique identifier for each fire (“Fire ID”), which was designed to capture unmeasured variation in suppression activity. All continuous predictors were centered and transformed to standard units to aid model fitting and facilitate comparison of effect sizes. Models were fit using the “speedglm” package (version 0.3‐5; Enea et al. 2015) in R.

Wildfire is a contagious spatial process—the likelihood of a pixel burning at high severity is strongly influenced by fire behavior in adjacent pixels. Therefore, fire severity data are often spatially autocorrelated, which artificially deflates standard error estimates. To account for this, we used spatial block bootstrapping to quantify parameter uncertainty (Lahiri 2018; see Appendix 3). Due to the computational intensity of this method, we took a 25% subsample of the data to speed model fitting. While block bootstrapping accounts for artificially small standard errors introduced by spatial autocorrelation, we recognize that including incoming severity as a covariate may introduce additional spatial information, potentially influencing estimates of other spatially structured predictors such as weather and forest structure.

#### 2.2.2 Machine Learning Methods to Understand the Importance of Forest Structure, Weather, Climate, and Topography

To compare the relative importance of forest structure, weather, climate, and topography for predicting fire severity, we trained two random forest classification models: one for forest structure quantified at the 90 m by 90 m (neighborhood) scale, and one for the larger 390 m by 390 m (stand) scale. Unlike regressions, random forest models do not presume an interaction structure or functional form for the relationships between predictors and outcomes (Breiman 2001). This makes them an ideal tool with which to examine the relative importance of various predictors.

To fit these models, we first centered all continuous predictors and scaled them to standard units (see Figure 3 for a complete list of predictors). The dataset was divided into a training dataset (80%) and a testing dataset (20%); but unlike the analysis of ownership described above, we did not further subsample the data. We used spatially blocked k‐fold cross validation on the training dataset to tune two key hyperparameters (maximum tree depth and number of subfeatures) while avoiding potential bias from spatial autocorrelation. We selected the best‐fitting model based on log‐loss, a measure of predictive accuracy. Finally, we used the testing dataset to evaluate the out‐of‐sample predictive ability of the best‐fitting model (Tables S3 and S4). Models were fit using the “MLJ” package (Blaom et al. 2020) in julia version 1.10.1 (Appendix 2; Bezanson et al. 2017). To compare the importance of each predictor, we quantified their Shapley importance values for a subset of 10,000 pixels (Sundararajan and Najmi 2020).

#### 2.2.3 Quantifying the Relationship Between Fire Severity and Its Drivers

The same flexibility that makes random forest models well‐suited for ranking the importance of predictors makes them poorly suited for understanding the functional relationships between predictors and outcomes. Therefore, to investigate the relationships between forest structure, weather, topography, and fire severity, we fit two binomial generalized linear models (GLMs), one for each spatial scale. These models include all the same variables as the random forest models, except that we now assume a linear relationship between these variables and the log‐odds of a pixel burning at high severity. To test the validity of this assumption, we compared the ranking of variables from the random forest models to the ranking of effect sizes in the GLMs. Similar rankings would suggest that the GLMs do not exclude key nonlinear relationships.

To investigate how the effects of forest management are modified under extreme weather conditions, we also included interactions between four of the five forest structure metrics and the hot‐dry‐windy index. We did not consider interactions between spatial homogeneity and the hot‐dry‐windy index due to high collinearity with stem density (Figures S11 and S12), which would complicate the interpretation of the interaction effect. As with the models fit to characterize the effect of ownership on the probability of high‐severity fire, we centered and standardized all continuous variables and estimated parameter uncertainty using spatial block bootstrapping. The data was again subsampled to 25% of its original size to reduce computation time.

#### 2.2.4 Comparing the Forest Structure of Private Industrial and Public Lands

Finally, we quantified the differences in forest structure found on private industrial versus public forestlands to determine whether they could explain disparities in high‐severity fire occurrence between these ownership types. To do so, we calculated the relative frequency of the values of each forest structure metric on the two ownership types using differenced density plots, which, though descriptive rather than analytical, allowed us to capture the complex differences across private industrial and public lands. We also generated similar plots for the non‐structural attributes discussed in this paper: weather, climate, and topography. All data and code used to conduct the analyses in this paper are publicly available (Levine et al. 2025a, 2025b).

#### 2.2.5 Sensitivity Analyses

We conducted five additional sensitivity analyses to assess whether our results were robust to key assumptions and modeling choices. (1), we tested whether our findings depended on the choice of severity metric by repeating the analyses using a binary metric derived from the delta normalized burn ratio (dNBR), rather than satellite‐estimated CBI. This analysis was motivated by two concerns: (i) satellite‐estimated CBI is not a direct field measurement but is inferred from satellite imagery using a machine learning model and (ii) that model includes climatic water deficit as a predictor, potentially introducing circularity in our analyses, which also include climatic water deficit as a covariate. (2), as a further test of circularity, we also refit each model after removing climatic water deficit as a predictor. We also tested the sensitivity of our findings to: (3) the level of subsampling, (4) the threshold CBI value for classifying fire severity, and (5) multicollinearity among forest structure metrics. While these analyses are too extensive to present fully in the main text, details are provided in Appendix S4.

## 3 Results

### 3.1 High‐Severity Fire Incidence Is Higher on Private Industrial Land

**FIGURE 2:**
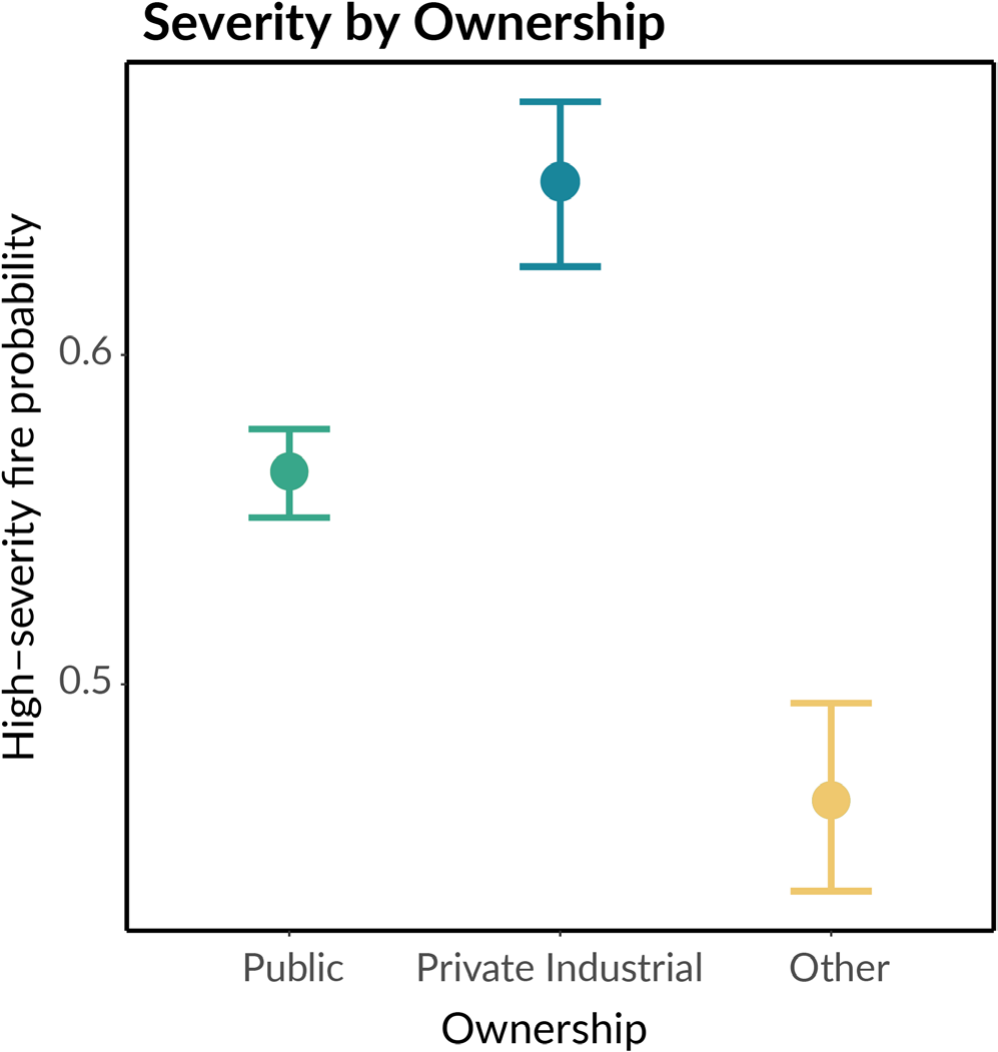
Fire severity by forest ownership. Estimated difference in the probability of a 30 × 30 m pixel burning at high severity after controlling for systematic differences in weather, climate, and topography across these ownership types. Points indicate the mean predictions of a binomial generalized linear model (Materials and Methods). Error bars indicate the upper and lower 95% confidence interval bounds, which were determined using spatial block bootstrapping to account for spatial autocorrelation in the underlying data. The “other” land ownership category corresponds to all pixels in the study area that were not owned by either private industry or public agencies (primarily small private landowners and non‐profit conservation organizations).

Private industrial ownership was associated with a 1.45 times increase in the odds of high‐severity fire compared to publicly owned land in the study area, and a 2.1 times increase compared to land owned by neither private industry nor public agencies (“other” land), even after controlling for the effects of both weather and biophysical characteristics like climatic water deficit, topographic position index, and slope (Figure 2). This corresponds to an increase in the probability of high‐severity fire occurrence of 9% over public land (65.7% [62.7%, 67.7%] vs. 56.5% [55.0%, 57.7%], brackets indicate bootstrapped 95% confidence intervals; see Materials and Methods and Table S2), and 19% over “other” land (46.5% [43.7%, 49.4%]), corroborating the results of previous analyses (Levine et al. 2022; Zald and Dunn 2018). The estimated effect of private industrial versus public land ownership was equivalent to a one standard deviation increase in the hot‐dry‐windy index, a three standard deviation decrease in fuel moisture, or a 10 standard deviation increase in heat load.

### 3.2 Both Forest Structure and Extreme Weather Are Important Predictors of High‐Severity Fire Occurrence

Both the neighborhood‐ and stand‐scale random forest models accurately predicted severity out of sample, with the stand‐scale model outperforming the neighborhood‐scale model by a small margin (AUC = 0.86 vs. 0.85; Tables S3 and S4). The models suggest that incoming severity, fire weather, and forest structure are each critical predictors of high‐severity fire occurrence.

**FIGURE 3:**
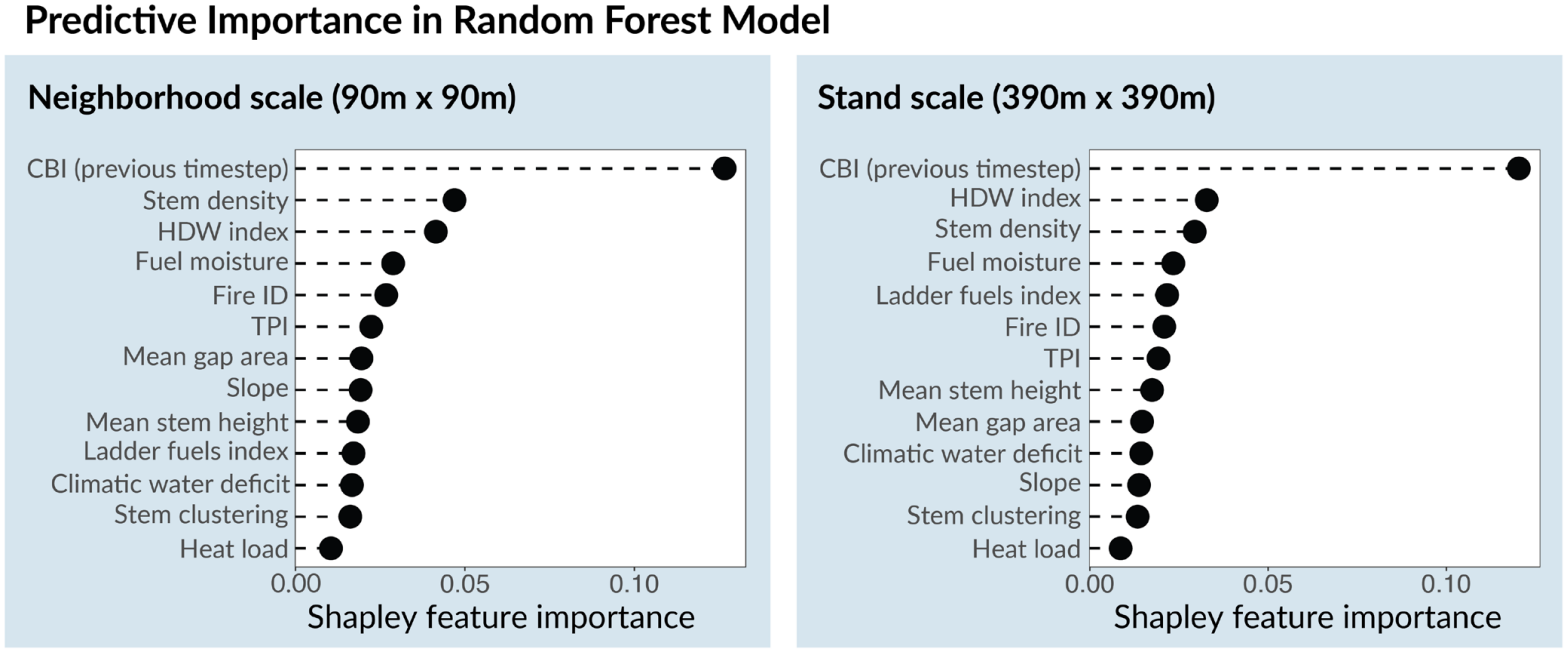
Variable importance in predictive models. Predictive variables in the random forest classification models ranked by Shapley feature importance. The left panel displays results for the neighborhood‐scale model, in which all forest structure metrics other than the ladder fuels index were calculated in a 90 m by 90 m box (~1 ha) centered on the focal pixel. The right panel displays results for the stand‐scale model, where structure metrics were calculated within a 390 m by 390 m box (~15 ha). To avoid potential bias, the pixels used to calculate feature importance were sampled evenly from private industrial and public land within the study area.

Incoming severity, a measure of a fire's effects as it approaches a given pixel, was the most important predictor in both models by a significant margin (Figure 3). This indicates that the contagious nature of fire—the propensity for high‐severity fire to drive further high‐severity effects—is the most important factor in predicting severity. For the neighborhood‐scale model, the second most important predictor was mean stem density, followed closely by the hot‐dry‐windy index. For the stand‐scale model, the hot‐dry‐windy index was the second most important predictor, followed by stem density, fuel moisture, and the ladder fuels index (Materials and Methods; Figure 3). Mean gap area, stem height, and topographic position index (TPI) were also important predictors of fire severity in both models (Figure 3). Overall, this analysis suggests that both weather and forest structure are key predictors of high‐severity fire occurrence.

### 3.3 Interactions Between Forest Structure and Weather Drive High Severity Fire Occurrence

Because the neighborhood‐ and stand‐scale models produced similar results, we focus on the stand‐scale model here, which had a lower AIC score (see Tables S5, S6 and Figure S6 for full results). Overall, the ranking of effect sizes in this model was very similar to the ranking of predictive importance from the random forest classifier, with only one difference among the seven most important variables (Figure 3; Table S5).

**FIGURE 4:**
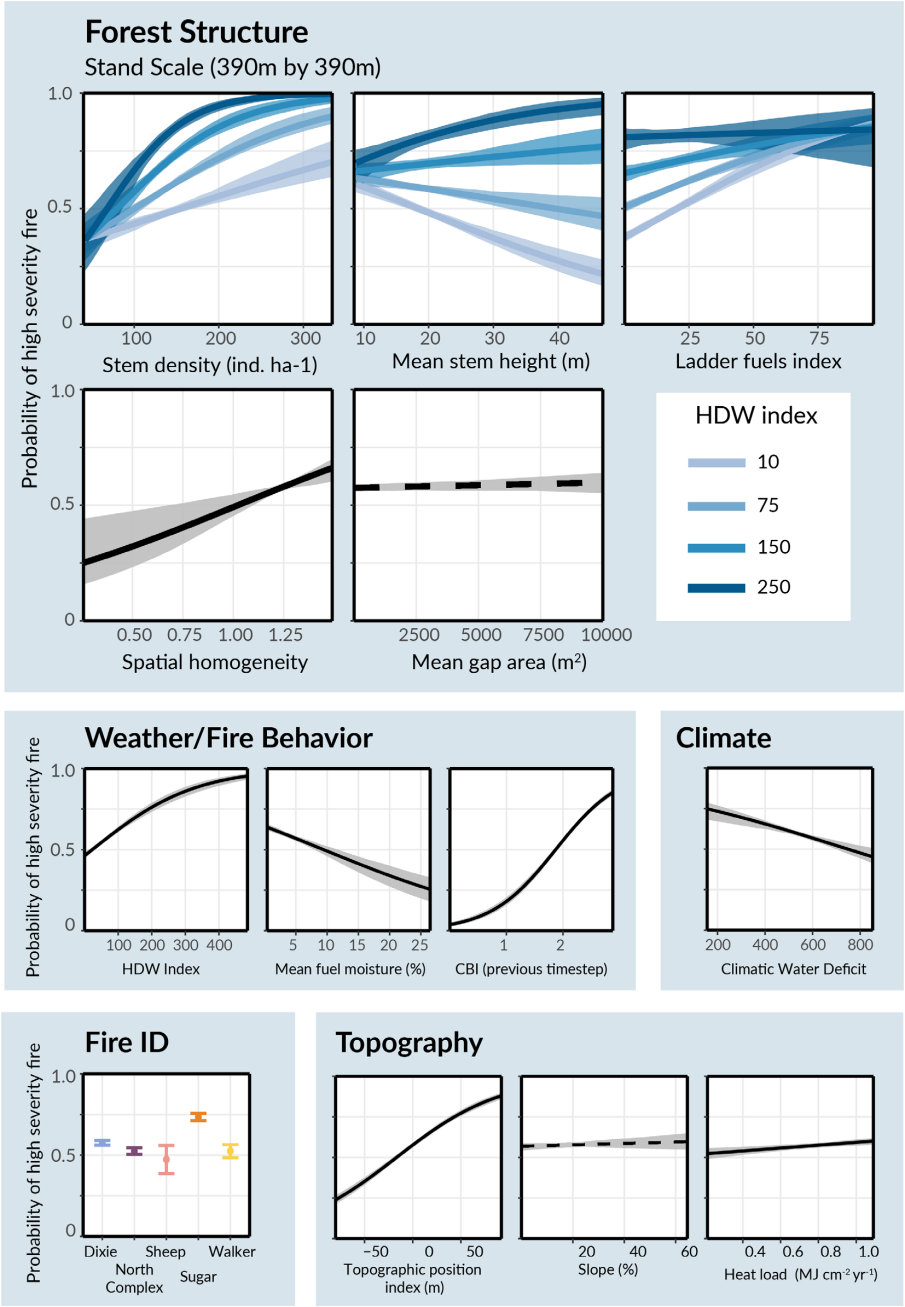
Effects of forest structure and weather on fire severity. Estimated effects of forest structure, weather, and topography on the probability of a 30 m by 30 m pixel burning at high‐severity. Shaded regions represent 95% confidence intervals, estimated using spatial block bootstrapping (Materials and Methods). Solid lines indicate a statistically clear relationship (95% CI does not contain 0), whereas dotted lines indicate the lack of a clear relationship. Darker blue colors illustrate the relationship under more extreme weather conditions (higher hot‐dry‐windy index). All relationships are shown for the stand scale (390 m by 390 m) model. Equivalent plots for the neighborhood scale model can be found in Figure S2.

We identified clear, positive effects of stem density (0.32 [0.25, 0.40]), ladder fuels index (0.21 [0.17, 0.24]), and spatial homogeneity (0.13 [0.06, 0.20]), and a clear, negative effect of mean stem height (−0.10 [−0.16, −0.04]) on the conditional probability of high‐severity fire (Figure 4; Table S6). The magnitude of these effects was large. A one standard deviation increase in stem density corresponded to a 1.4 times increase in the odds of high‐severity fire occurrence, equivalent to the effect of a 1.5 standard deviation increase in topographic position index, a 2.5 standard deviation decrease in fuel moisture, or a 6.4 standard deviation increase in heat load. The effects of ladder fuels, homogeneity, and stem height were smaller, but still substantial relative to the effects of topographic variables known to influence fire severity (Figure 4). Together, these results suggest that dense, homogeneous stands with high ladder fuels and small trees were more susceptible to high‐severity fire.

Both weather metrics had clear effects on high‐severity fire occurrence (Figure 4). The probability of high‐severity fire increased with the hot‐dry‐windy index (0.41 [0.35, 0.46]) and decreased with fuel moisture (−0.13 [−0.17, −0.09]) (Figure 4). A one standard deviation increase in the hot‐dry‐windy index corresponded to a 1.5 times increase in the odds of high‐severity fire, similar to the effect of stem density. The effect of fuel moisture was smaller, but similar in magnitude to the effects of spatial homogeneity and stem height (Figure 4; Table S6). Incoming severity, topographic position index, heat load, and climatic water deficit also exhibited clear relationships with severity.

Weather modified the effects of several forest structure metrics on severity, as evidenced by significant interactions between hot‐dry‐windy index and stem density, average stem height, and ladder fuels index (Figure 4). The interaction between hot‐dry‐windy index and stem density was positive (0.15, [0.098, 0.20]), meaning density‐driven increases in the probability of high severity fire were magnified in extreme weather conditions (Figure 4). The clear, positive interaction between hot‐dry‐windy index and stem height (0.15 [0.11, 0.20]) suggests that extreme weather could reverse the moderating effect of tree size on fire severity. In milder weather conditions, larger trees were associated with reduced probability of high‐severity fire occurrence. But in extreme conditions, the presence of large trees increased the probability of high‐severity fire (Figure 4). Finally, there was a negative interaction between hot‐dry‐windy index and ladder fuels index (−0.063 [−0.10, −0.021]), meaning the effect of ladder fuels on fire severity was weaker in extreme conditions.

### 3.4 Forest Structures Found on Private Industrial Land Are Correlated With High‐Severity Fire

**FIGURE 5:**
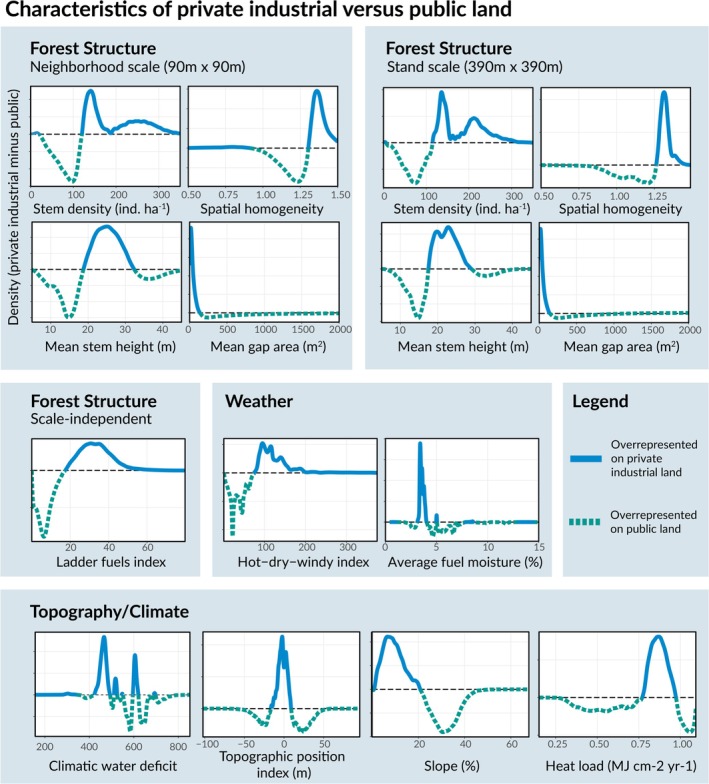
Effects of forest structure and weather on fire severity. Density difference plots characterizing the distribution of key forest structure, weather, and topographic variables on public versus private industrial forestland. Plots were generated by first subsampling the data such that there was an equal number of datapoints from each ownership class. Then, the density of each variable was calculated for each ownership class and then differenced to generate the panels shown in the figure. When the density difference is below the dotted line (< 0), it indicates that a value is overrepresented on public land (green lines). When the density difference is above the dotted line, it indicates that a value more prevalent on private industrial land (blue lines).

In general, private industrial land in the study area was characterized by forest structures associated with increased high‐severity fire risk (Figure 5). Compared to public lands in the study area, a larger proportion of private industrial lands had high stem density, homogeneous tree spacing, and more ladder fuels at both the neighborhood and stand scales (Figure 5). These three metrics have the largest estimated effect on high‐severity fire probability out of all forest structure variables (Figure 4), meaning discrepancies between public and private industrial forestland in these metrics could help explain the increased severity observed in industrial forests. Private industrial forests also experienced more extreme fire weather, both in terms of fuel moisture and hot‐dry‐windy index (Figure 5). Likely this is because private industrial forests are typically found on low–mid elevation sites (Figure 5). While the prevalence of extreme weather conditions certainly contributes to elevated fire severity in industrially managed forests, models which account for these differences still estimate that the probability of high‐severity fire is elevated on industrial forestland (Figure 2).

### 3.5 Sensitivity Analyses

The results of our study were robust to the choice of severity metric, and showed no evidence for circularity introduced by the inclusion of climatic water deficit as a model covariate (Appendix 4, Tables S8–S13). Replacing satellite‐estimated CBI with dNBR did not cause the estimated effects of ownership, weather, or forest structure to change sign or for their 95% confidence intervals to overlap zero (Tables S8–S10). The results were similarly unaffected by removing climatic water deficit as a covariate (Tables S11–S13). Additionally, the qualitative results of our study were insensitive to variation in the level of subsampling (Figures S7 and S9), the choice of CBI threshold for delineating fire severity (Figures S8 and S10), and multicollinearity among forest structure metrics (Figure S13). See Appendix 4 for more details on these analyses.

## 4 Discussion

In this study we established the clear importance of forest structure in driving wildfire severity—even under the extreme burning conditions associated with very large wildfires (> 100,000 ha)—and leveraged this finding to explain observed increases in severity in industrial forests. Metrics describing forest structure, in particular stem density and spatial homogeneity, were consistently ranked among the most important predictors of high‐severity fire (Figure 3). The predictive importance of stem density was comparable to that of the hot‐dry‐windy index, and consistently higher than fuel moisture, two weather metrics known to drive extreme fire behavior (Figure 3). On both public and privately owned lands, stands characterized by high tree densities, homogeneous spatial structure, and more continuous ladder fuels were more likely to burn at high severity. Moreover, extreme weather magnified the effect of stem density on fire severity, suggesting that interventions which remove live trees are critical for moderating severity under climate change.

### 4.1 Patterns in Fire Severity Across Ownerships

We observed a clear increase in the probability of high‐severity fire occurrence on private industrial land relative to land managed by public agencies (Figure 2), echoing the findings of prior studies (Bousfield et al. 2025; Levine et al. 2022; Zald and Dunn 2018). This is true even after controlling for differences in topography, climate, and weather between these ownership types, suggesting that the management practices of private industrial timber companies contribute to the greater frequency of high‐severity fire on these lands. The probability of high‐severity fire was lowest on land owned by neither private industry nor the public, that is, “other” land (Figure 2). However, this third category was significantly smaller than the other two and contains a large diversity of landowners, including single‐family homes, small private landowners, ranches, and private conservation lands. Additionally, these properties tend to be closer to roads and population centers, meaning they have increased access to suppression resources which could partially explain their relatively low severity (Figure 1; Levine et al. 2022).

Our analysis provides a data‐driven explanation for observed increases in the probability of high‐severity fire on private industrial timberland relative to publicly managed forests. A higher proportion of private industrial forestlands in our study area were characterized by dense, spatially homogeneous stands with high amounts of ladder fuels. It is possible, if not likely, that these dense, homogeneous forest conditions are a product of conventional forest management practices tied to timber objectives. These practices include even‐aged silviculture with 60–80‐year rotations, maximizing growing space occupancy for “crop” trees, and minimal non‐commercial intermediate stand treatments (i.e., pre‐commercial or fuels reduction thinning, mastication, or prescribed fire). The forest structural attributes associated with these practices (also see Stephens and Moghaddas 2005), in combination with severe fire weather, were strongly associated with increased probability of high‐severity fire (Figures 4 and 5). Together, these results suggest conventional forest management practices that are often characteristic of industrial forests could be modified to reduce the likelihood of high‐severity fire effects.

The proportion of high‐severity fire effects, on both public and private industrial forest land in our study area, was considerably higher than historical estimates for these forest types (Safford and Stevens 2017) and continues to increase despite ownership (Miller et al. 2012; Parks et al. 2016; Steel et al. 2018; Stevens et al. 2017). Within our study area, the probability of burning at high severity was high on both public and private lands, ranging from a mean of 56.5% on public land to 65.7% in private industrial forests. Thus, the results of this study should not be taken as evidence that forest management practices conducted on public lands are effective at reducing fire severity. Extensive forest restoration and fuel reduction treatments are necessary to mitigate fire severity across a majority of California's frequent‐fire adapted forests (Stephens et al. 2024), especially given forecast increases in extreme fire weather under climate change (Abatzoglou and Williams 2016; Little Hoover Commission 2018). This study provides key information about specific forest structure metrics that should be targeted for doing so.

### 4.2 The Interactive Effects of Forest Structure and Extreme Weather on Fire Severity

Metrics describing forest structure (stem density, spatial homogeneity, and ladder fuels index), weather (hot‐dry‐windy index and fuel moisture), and incoming severity were consistently ranked as the most important predictors of high‐severity fire in random forest models at both spatial scales examined and across ownerships (Figure 3). On all ownership types, dense, homogeneous forest stands with high ladder fuels were more likely to burn at high severity, mirroring findings from prior studies that indicate an important role of both heterogeneity and total fuel loads in driving extreme fire effects (Forbes et al. 2022; Hakkenberg et al. 2024; Kane et al. 2015; Koontz et al. 2020; Steel et al. 2021). The probability of high‐severity fire also increased with more severe weather conditions (i.e., increasing hot‐dry‐windy index and lower fuel moistures).

The consistent ranking of both weather and structural metrics as key predictors of fire severity is an important revelation in light of concerns that extreme weather might overwhelm efforts to mitigate fire severity through appropriate forest management (Bradstock et al. 2010; Cary et al. 2009; Penman et al. 2013). To the contrary, this study suggests that management interventions which alter forest structure, particularly by reducing tree density and spatial homogeneity, may be highly effective tools of severity mitigation even in extreme weather conditions (Figure 4). This corroborates prior studies of fuel treatment efficacy in single fire events and recent meta‐analyses which have found a clear, mitigating effect of thinning and other fuel reduction practices on fire severity (Davis et al. 2024; Lydersen et al. 2017; Lyons‐Tinsley and Peterson 2012; Prichard et al. 2020).

Weather was found to modify the effects of several key forest structure attributes on high‐severity fire occurrence, providing important insight into which management interventions may be most effective in a warmer future. For high values of the hot‐dry‐windy index, the effect of stem density on the probability of high‐severity fire was magnified. This indicates that management interventions that reduce tree density are even more critical under extreme weather conditions (Figure 4). Similarly, extreme weather appeared to reverse the moderating effect of stem height on the probability of high‐severity fire occurrence at both spatial scales (Figures 4 and S6). Possibly, extreme fire behavior driven by high winds and hot temperatures overwhelms the greater fire resilience of large trees, instead turning them into a fuel source which further exacerbates these extreme dynamics. Finally, the correlation between high ladder fuels index and high‐severity fire incidence was weaker under extreme weather conditions. In hot, windy, and dry conditions vertical fuel connectivity, which can vector fire into the canopy, may be less important due to the increasing likelihood of existing crown‐to‐crown fire transmission.

### 4.3 Management Implications

The clear interaction between tree density and the hot‐dry‐windy index underscores the importance of effective fuel and forest management, especially in a warmer climate. For a hot‐dry‐windy index of 150, our model predicts that a decrease in LiDAR‐estimated stem density from 200 to 100 trees per hectare results in a 28% decline in the probability of high‐severity fire (85% [82%, 89%] vs. 57% [53%, 61%]). Even in mild weather conditions, stem density has a large estimated effect on the probability of high‐severity fire. For a hot‐dry‐windy index of 50, a decline from 200 to 100 trees per hectare was associated with an 18% decline in the probability of high‐severity fire (66% [64%, 70%] vs. 47% [45%, 49%]).

This finding—that the effect of density is magnified under severe weather—is critical and suggests that to moderate extreme fire effects, managers should focus on reducing tree density (Schwilk et al. 2009). Prescribed fire, which is often preferred over mechanical thinning by environmental groups due to its reduced impact, does not typically reduce the density of medium and large trees (Knapp et al. 2017; Schwilk et al. 2009). Instead, the goal of prescribed fire is to reduce surface fuels and the density of small trees (ladder fuels) (Ryan et al. 2013). We did not quantify surface fuels in our analysis because of LiDAR data limitations and are thus unable to provide information about the full efficacy of treatments, like prescribed fire, that focus on reducing these fuels. However, previous studies have demonstrated that reducing surface fuels is key to reducing wildfire intensity and tree mortality in western United States frequent‐fire adapted forests (Davis et al. 2024; Shive et al. 2024; Stephens et al. 2009). Indeed, there is increasing evidence that combined thinning and prescribed fire treatments are most effective at mitigating fire severity (Bernal et al. 2025). A recent paper from the southern Cascade Mountains (just north of our study area) found that mechanical thinning treatments that reduced overstory tree density in conjunction with prescribed burns were most effective at limiting crown fire behavior in a severe wildfire (Brodie et al. 2024). Similarly, a meta‐analysis of fuel treatment effects on subsequent fire severity found that the most effective strategy was combined overstory thinning and prescribed fire (Davis et al. 2024). These findings, in combination with the results of our study, suggest that effective severity mitigation strategies should involve treatments that reduce both overall tree density and surface fuels.

The most important driver of high‐severity fire occurrence in each analysis was incoming severity, the average satellite‐estimated CBI of pixels which burned in the preceding 8‐h interval (Figures 3 and 4). This result emphasizes the contagious nature of wildfire: a fire burning at high severity tends to continue burning at high severity. Likewise, activities which reduce fire severity in one location are likely to have important downstream effects, reducing fire severity in areas that burn immediately after. An important implication of this finding is that forest structure is not only important at the neighborhood or stand scales quantified in this study, but also at the landscape scale, indicating that cross‐ownership partnerships and regional planning are necessary to effectively reduce fire severity (Little Hoover Commission 2018). Indeed, prior studies have found similar landscape‐scale effects of fuel reduction treatments, forest structure, and past fire behavior (Chamberlain et al. 2024; Lydersen et al. 2017; Povak et al. 2020; Urza et al. 2023).

## 5 Limitations

This study is unique in the availability of extensive, high‐resolution spatial data that provided a snapshot of vegetation structure prior to the arrival of several large wildfires, allowing us to examine the mechanisms underlying increases in fire severity. However, there are several limitations with this analysis. First, while substantial efforts were made to quantify fire weather at a similar resolution and scale to the LiDAR‐derived forest structure data, weather data is inherently less precise. We employed a sophisticated interpolation of weather from numerous remote weather stations (RAWS; Figure S1), applying a novel algorithm to delineate the temporal progression of each fire from infrared satellite imagery (Materials and Methods). While this approach makes the analysis well‐suited to compare the importance of weather, forest structure, ownership, and other variables compared to prior studies of fire severity, the rankings of weather and forest structure's effects should not be overinterpreted.

Second, although satellite‐estimated CBI is highly correlated with empirical mortality assessments (Lydersen et al. 2016; Miller et al. 2009), it is ultimately an indirect measure of fire severity (Parks et al. 2019). This has three key implications. The first is that satellite‐estimated CBI cannot differentiate between young stands that burned at high severity and old stands that burned at high severity, scenarios with distinct implications for carbon, timber loss, and wildlife habitat. The second is that satellite‐estimated CBI does not distinguish between mortality caused by wildfire and mortality caused by post‐fire salvage logging, a common practice on industrial forestland. While this has the potential to bias estimates of relative severity on private industrial versus public land (Safford et al. 2015), a prior study found that the size of this bias is small, even in fires where extensive salvage logging took place (Levine et al. 2022). Likely, this is because stands targeted for salvage logging are those which experienced high‐severity fire effects. Finally, because the random forest model used to estimate CBI from satellite imagery incorporates climatic water deficit as a predictor, there is a potential for circularity in our analyses, which also include climatic water deficit as a covariate. However, sensitivity analyses indicate that circularity did not impact the results of this study, which were robust to the choice of severity metric (dNBR vs. CBI; Appendix 4, Tables S8–S10), and the inclusion of climatic water deficit as a covariate (Appendix 4, Tables S11–S13).

The third key limitation of this study is that LiDAR often under‐detects smaller trees, especially under dense, multi‐layered tree canopies (Jeronimo et al. 2018). This likely biases our estimates of stem density and the ladder fuels metric in areas with high canopy cover. However, it is unlikely that this substantially impacted the outcome of the study given (i) our analysis was comparative, and therefore does not rely on absolute estimates of density or ladder fuels and (ii) lidar‐derived stem density, despite being an underestimate of true density, had a clear relationship with severity. The final limitation is that while LiDAR allows us to quantify pre‐fire forest structure at the individual tree scale, our estimates of fire severity, and therefore mortality, are at the larger 30 m by 30 m scale of Landsat satellite imagery. This mismatch in scale precludes us from capturing fine‐scale changes in forest structure, which could have important impacts on subsequent ecosystem dynamics, for example if single seed‐bearing trees remain even in some high‐severity patches. This suggests that efforts to gather post‐fire LiDAR data would be highly valuable for future research.

## 6 Conclusions

Coincident with rapid anthropogenic climate change, high‐severity fire occurrence is increasing across California and the western United States, a trend that poses significant threats to socioeconomic and ecological systems including timber production, key wildlife habitat, human health and safety, and natural climate solutions (Anderegg et al. 2020; Bousfield et al. 2023; Burke et al. 2021; Davis et al. 2019; Jones et al. 2016; Reid et al. 2016). Understanding the drivers of fire severity is thus critical for mitigating their worst impacts in an uncertain future.

Here we discovered that the characteristics of private industrial forestland are strongly correlated with increases in the probability of high‐severity fire, potentially explaining the higher incidence of high severity fire in these forests (Bousfield et al. 2025; Levine et al. 2022; Zald and Dunn 2018). Evidence that forest structure has a similar effect on high‐severity fire occurrence as extreme weather, and that these effects are magnified under extreme weather conditions, suggests potential changes in current forest management practices are needed to mitigate fire severity in a warmer future, regardless of ownership. Specifically, treatments that reduce stand density, increase spatial heterogeneity, and reduce vertical fuel continuity and loads (ladder fuels) may be effective at slowing increases in fire severity in California and the western USA.

The state of California and the US Forest Service, Pacific Southwest Region, recently released a plan outlining a shared goal of significantly increasing the annual rate of forest restoration and fuel reduction treatments to address the current wildfire problem (California Governor's Forest Management Task Force 2021). While there are many impediments to reaching the stated goal of treating 400,000 ha annually (Clark et al. 2024), perhaps in the wake of recent record‐breaking fire years and emerging scientific consensus on the benefits of thinning and prescribed fire (Brodie et al. 2024; Cova et al. 2023; Davis et al. 2024; Safford et al. 2022) there will be enough momentum to overcome these impediments across both private and public forestlands.

## Author Contributions

**Jacob I. Levine:** conceptualization, data curation, formal analysis, investigation, methodology, project administration, software, visualization, writing – original draft, writing – review and editing. **Brandon M. Collins:** conceptualization, data curation, methodology, project administration, supervision, writing – review and editing. **Michelle Coppoletta:** conceptualization, data curation, methodology, resources, writing – review and editing. **Scott L. Stephens:** conceptualization, funding acquisition, project administration, supervision, writing – review and editing.

## Conflicts of Interest

The authors declare no conflicts of interest.

## Supporting information


Data S1.

## Data Availability

The data and code that support the findings of this study are openly available in Zenodo at https://doi.org/10.5281/zenodo.15848846 and https://doi.org/10.5281/zenodo.15850340, respectively, as well as Github at https://github.com/jsilevine/severity_silviculture. Meteorological data were obtained from the Western Regional Climate Center at https://raws.dri.edu. Climatic water deficit data were obtained from the US Geological Survey (USGS) at https://doi.org/10.5066/P9PT36UI (version 5.0). Digital elevation model data were obtained from the U.S. Geological Survey Science Data Catalog (SDC) at https://data.usgs.gov/datacatalog/data/USGS:35f9c4d4‐b113‐4c8d‐8691‐47c428c29a5b. Landsat data were obtained from the U.S. Geological Survey at https://doi.org/10.5066/P975CC9B, https://doi.org/10.5066/P9TU80IG, and https://doi.org/10.5066/P918ROHC. Fire perimeter data were obtained from CalFire's Fire and Resource Assessment Program at https://www.fire.ca.gov/what‐we‐do/fire‐resource‐assessment‐program/fire‐perimeters. Heat incidence data derived from Suomi VIIRS data were obtained from NASA's Earth Science Data and Information System at https://doi.org/10.5067/FIRMS/VIIRS/VNP14IMGT_NRT.002. Due to large file size, LiDAR data are available on request.
