# Supplementary material for: Extreme Weather Magnifies the Effects of Forest Structure on Wildfire, Driving Increased Severity in Industrial Forests
Source: Glob Chang Biol. 2025 Aug 20;31(8):e70400. doi: 10.1111/gcb.70400 (PMC12365866; doi:10.1111/gcb.70400)
Supplement: Supplementary file 1 — Data S1. [file GCB-31-e70400-s001.pdf]

Supplementary Materials for

Extreme weather magnifies the effects of forest structure on wildfire, driving increased severity in industrial forests

Jacob I. Levine, Brandon M. Collins, Michelle Coppoletta, Scott L. Stephens

Appendices

SI Appendix 1. Algorithm to establish fire progressions from infrared satellite imagery.

To estimate the temporal progression of fires in our study area we developed an algorithm based on the methodology described in (Briones-Herrera *et al.* 2020) and (Stephens *et al.* 2022) to detect fire perimeters from visible infrared imaging radiometer suite (VIIRS) imagery taken onboard NASA's Suomi National Polar-Orbiting Partnership spacecraft.

The first step is to establish final fire perimeters, which we did using CalFire's Fire and Resource Assessment Program Historical Fire Perimeter data. These data record the initial alarm date and containment date, which we use to bound the fire progression data. Using this information we then deploy the following algorithm to delineate the temporal progression of each fire:

1. Subset the VIIRS data to the spatial and temporal extent of the final fire perimeter.
2. Identify all unique timepoints in the VIIRS dataset that fall between the alarm and containment dates. We label these timepoints t_i, where i indexes each unique timepoint.
3. For each unique timepoint, t_i:
   i. Subset the VIIRS data, V, to set v such that all $t = t_i$.
   ii. Use a spatial clustering algorithm to separate v into contiguous chunks: C. Here we use the functions 'hclust' and 'cutree' in R version 4.4.1 (R Core Team 2013). We set the minimum distance for cutting the tree generated by 'hclust' (the parameter 'h' in 'cutree') to 1125m.
   iii. Remove all clusters with less than Q total VIIRS observations, where Q sets a tolerance threshold to avoid including data from non-fire infrared sources. For this analysis we set $Q = 4$.
   iv. For each set of VIIRS observations in C, determine the convex hull that contains each point. We used the package 'concaveman' (Gombin *et al.* 2020) in R, setting the concavity value to 3.
   v. We determine the intersection of the hulls calculated in (iv) and the final perimeter, to avoid falsely including unburned areas outside the final fire perimeter. The assume the union of these hulls to be the fire perimeter at time t_i.
   vi. We subtract the fire perimeter at time t_{i-1} from the perimeter at time t_i to determine the area that burned between each timestep. We perform these calculations using the 'sf' package (Pebesma & Bivand 2023) in R.
4. After calculating the new burned area for each timestep, we rasterize the resulting shapefiles to obtain a grid of burn times using the 'terra' package (Hijmans *et al.* 2022) in R.
5. To downscale the estimates from the VIIRS timesteps to a regular 8-hour progression on a 30m-by-30m grid, we use moving-window averaging to estimate the time at which each pixel burned. Specifically, for each pixel we calculate the average burn-time of all pixels in a 750m by 750m box centered on the pixel. Then, we group these into regular 8-hour bins defined by: 24:00-7:59, 8:00-15:59, 16:00-23:59 hours.

SI Appendix 2. Random forest classification models: specification and hyperparameter tuning

To evaluate the relative importance of forest structure, weather, climate, and topography for predicting high severity fire occurrence we fit two random forest classification models, one for each spatial scale. Aside from the spatial scale of the forest structure metrics, the two models were identical, including the following predictive features: mean stem density, spatial homogeneity, mean gap area, mean stem height, ladder fuels index, climatic water deficit, slope, topographic position index, heat load, incoming severity (mean CBI in previous timestep), fuel moisture, hot-dry-windy index, and fire name.

To fit the models, we first tuned two key hyperparameters, maximum tree depth and the number of subfeatures, using a grid search and k-fold cross validation. We first grouped the data into contiguous blocks based on the scale of autocorrelation estimated from the residuals of the binomial GLMs (see SI Appendix 3 for a description of how this was estimated). Then, we separated the data into a training dataset (80%) and testing dataset (20%) by dividing the contiguous blocks amongst these two groups. We divided the data using these blocks to maintain the spatial structure and thereby limit the influence of spatial autocorrelation on the model tuning and testing procedures. We then further divided the training dataset into 10 folds, again maintaining the spatial structure within blocks, to use in cross-validation. For each unique combination of hyperparameters, we fit the model withholding 1 of the 10 folds and evaluated the log-loss when predicting the withheld fold. By averaging across the 10 folds, we obtained an estimate of the predictive performance of the model with each combination of hyperparameters.

We selected the set of hyperparameters with the lowest log-loss value, and then fit the model to the full training dataset. Finally, we evaluated the predictive performance of the tuned model on the testing dataset, using area-under-the-curve (AUC) and confusion matrices to assess accuracy and recall. The models were fit in the julia programming language (Bezanson *et al.* 2017) using the machine learning in Julia (MLJ) library (Blaom *et al.* 2020).

SI Appendix 3. Model Specification and Spatial Block Bootstrapping Methodology.

4.1 Model specification

To describe the relationship between ownership, forest structure, weather, climate, topography and the probability of high severity fire we fit binomial generalized linear models (GLMs) of the following form:

$$logit(E[Y]) = \beta X$$
$$Y \sim Binomial(E[Y])$$

where $Y = \begin{bmatrix} y_1 \\ \vdots \\ y_N \end{bmatrix}$ is the vector of binomial response variables indicating whether each pixel burned at high severity ($y_1 = 1$ when the pixel burned at high severity, and $y_1 = 0$ otherwise). $\beta = \begin{bmatrix} \beta_0 \\ \vdots \\ \beta_Q \end{bmatrix}$ is the vector of parameters to be estimated, and X is the NxQ model matrix. For a full list of the parameters estimated in each model, see Figures S2, S5, and S6. All models were fit using the 'speedglm' package (Enea *et al.* 2015) in R.

4.2 Spatial Block Bootstrapping

To eliminate bias in standard error estimates introduced by spatial autocorrelation in our data, we performed a spatial block bootstrapping routine as described by (Lahiri 2018), and implemented in (Levine *et al.* 2022). The algorithm is as follows:

1. Fit a naïve generalized linear model (GLM) on the full dataset. The main effect parameter estimates reported in the paper are taken from this model, whereas the standard errors for these estimates are calculated using spatial block bootstrapping.
2. Fit a spherical semi-variogram to the residuals from the naïve model to determine the scale of spatial autocorrelation for each model. This value, which we label b, defines the block size in subsequent steps.
3. Define the minimum set of non-overlapping blocks, B, of size bxb, whose union covers the entire study area, A. We label the number of blocks required to cover A, N.
4. For each bootstrap iteration (we use 200): generate a random sample, k, of N points such that for each point a block of size bxb centered on that point is contained fully within the union of B.
5. The blocks centered on k become the bootstrap blocks K.
6. Extract all data that fall within K (sampling with replacement when the extent of the blocks overlap).
7. Fit a new GLM on this data and record the parameter estimates.

After all iterations are complete, the variation in parameter estimates generated in step 7 can be used to calculate the standard errors and confidence intervals for each parameter. All models

were fit using the package ‘speedglm’ in R (Enea *et al.* 2015), and the semivariograms were fit using the package ‘gstat,’ also in R (Gräler *et al.* 2016; Pebesma 2004).

SI Appendix 4. Sensitivity Analyses.

4.1 Choice of severity metric (dNBR vs. CBI)

Throughout the paper we quantify fire severity using the composite burn index (CBI), which we estimate from Landsat imagery using the random forest classification algorithm described in Parks *et al.* (2019). We chose this metric for two primary reasons. First, it has an established correlation to on-the-ground measurements of live basal area mortality, the ecological outcome we are ultimately interested in (Lydersen *et al.* 2016). Second, this paper is in many ways a follow-up to Levine *et al.* (2022), a paper where we establish that the probability of high-severity fire occurrence, as quantified using CBI, is higher on private industrial than public land. Because our goal in this paper was to: (a) understand whether this pattern holds in recent, more extreme 'mega-fires', and (b) identify the mechanisms causing severity to increase on private industrial land, we elected to use the same method of quantifying fire severity. The results of the two papers are therefore directly comparable.

However, we recognize that using satellite-estimated CBI has several key drawbacks: 1) it is an indirect measure of fire severity, quantified using a machine learning model trained on Landsat satellite imagery and field observations of CBI; and 2) the model we use to estimate CBI includes climatic water deficit as a predictor, introducing potential circularity into our analyses, which also include climatic water deficit as a covariate. To evaluate the sensitivity of our results to the choice of satellite-estimated CBI as a metric of fire severity, we refit each of the three generalized linear models described in the main text (ownership, neighborhood-scale forest structure, and stand-scale forest structure) using a binary severity metric derived from dNBR instead of CBI. We again used spatial block bootstrapping to estimate robust standard errors and 95% confidence intervals. However, we only used 100 bootstrap iterations to reduce computation time.

Unlike CBI, dNBR is a metric calculated directly from Landsat satellite imagery using a fixed equation rather than estimated using a machine-learning algorithm trained from field observations (Miller & Thode 2007). As with CBI, there is an established threshold value of dNBR that is used to discriminate between high- and low/moderate-severity fire effects (367; Miller & Thode 2007). Using dNBR and its associated threshold to categorize pixels by severity resulted in minor changes to the categorization of pixels in the study area. The classification of 298,272 pixels were changed when using dNBR (roughly 9% of the overall dataset). Of the pixels which changed status, most (242,325; 7.3%) shifted from low-moderate to high-severity.

Refitting the models using the dNBR-derived severity classification resulted in minimal changes to the estimated coefficients (Tables S8-10). Private industrial forests were still the most likely to experience high-severity fire effects, while "other" lands were the least likely. Using dNBR magnified these differences slightly, increasing the effect of private industrial relative to public ownership (Table S8). The only notable change in the size, sign, or clarity of other coefficients in the ownership model, was the reduction in the strength of average fuel moisture's effect from -0.12 to -0.03.

Similarly, the models of forest structure exhibited only small shifts in the sign and statistical clarity of estimated coefficients at both the neighborhood and stand scale, and the central results were unchanged (Tables S9-10). For the neighborhood-scale model, notable changes were: (i) the magnitude of fuel moisture's effect decreased; (ii) the effect of slope increased in magnitude and became statistically clear (i.e. 95% CI no longer overlapped zero); and (iii) the interaction

between mean gap area and the hot-dry-windy index became statistically unclear (Table S9). For the stand-scale model, the notable changes were: (i) the effect of fuel moisture declined in magnitude; (ii) the effect of mean gap area became statistically clear, although the corresponding increase in effect magnitude was small (0.003); and (iii) the effect of slope increased in magnitude and became statistically clear (Table S10).

Notably, none of the changes in either the ownership or forest structure models involve the main results of the paper, indicating that those results are robust to the choice of severity metric. In particular, the probability of high severity fire is still highest on private industrial land, that pattern can plausibly be explained by the presence of dense and spatially homogenous forests on forests owned by private industry, and extreme weather magnifies the effects of forest structure on fire severity. Additionally, there were no substantial shifts in the effect of climatic water deficit in any of the models, suggesting that circularity was not a major factor driving the results of models fit using CBI-derived classifications of fire severity.

4.2 Circularity in models with climatic water deficit as a covariate

As an additional test for circularity in models of CBI-derived fire severity classification that include climatic water deficit as a covariate, we again refit each of the generalized linear models from the main text after removing climatic water deficit as a covariate. This resulted in no discernible changes to the results (Tables S11-13). The shifts in model coefficients, where observed, were universally small, and the statistical clarity of each effect was unchanged (Tables S11-13). The fact that none of the estimated effects changed after removing climatic water deficit suggests that circularity did not play a significant role in the results reported in the main text.

As a final test of the role of circularity, we directly quantified the predictive importance of climatic water deficit. If there were substantial impacts of circularity, then climatic water deficit would likely be an important predictor of the CBI-derived severity classification. To determine climatic water deficit's predictive importance, we refit the stand-scale random forest classification algorithm after removing climatic water deficit as a predictive feature and quantified the change in out-of-sample predictive performance. The change in AUC after excluding climatic water deficit was small (0.006), indicating that climatic water deficit is not an important predictor of the CBI-derived severity classification.

In summary, three lines of evidence suggest that the results of this study are robust to circularity introduced by including climatic water deficit as a covariate: (i) using dNBR instead of CBI did not substantially alter the estimated effect of climatic water deficit on the probability of high-severity fire (section 4.1); (ii) removing climatic water deficit as a predictor did not impact the estimated effects of other covariates; and (iii) climatic water deficit is not an important predictor of high-severity fire occurrence in the random forest classification models.

4.3 Level of subsampling in block-bootstrapped analyses

Spatial block bootstrapping is computationally cumbersome – it requires generating hundreds of unique, randomly sampled and spatially structured datasets and refitting the model on each of these to estimate robust standard errors. Therefore, to make our analyses computationally feasible, we subsampled our dataset to 25% its original size before conducting the spatial block bootstrapping routine. To test whether our results were sensitive to the chosen level of subsampling (25%), we refit each of the generalized linear models described in the main text to

new data subsets generated across a range of subsampling intensities, tracking how the estimated coefficients changed. While the computational demands of block bootstrapping precluded us from re-estimating standard errors at each level of subsampling, we are confident that the qualitative results of our analysis are unlikely to change without large shifts in the mean coefficient estimates. Such shifts would be apparent in this analysis.

Despite varying the subsampling level from 2% to 62%, we observed minimal changes in the coefficients of both the stand- and neighborhood-scale models of forest structure's effect on fire severity (Fig. S7 and S9). The only coefficients that changed substantially were the estimated mean effects of fire ID for the Sheep and Walker fires, and only for levels of subsampling below 20%. This was expected, because the Sheep and Walker fires are the two smallest fires in our dataset, meaning they are most susceptible to random variation across subsamples. Granted, shifts in the estimated effects of Fire ID do not affect the main takeaways of the paper. Therefore, we conclude that our results are robust to the choice of subsampling intensity.

4.4 Threshold for high versus not-high severity

To distinguish high-severity fire effects in our dataset we used a threshold value of CBI established from empirical data (CBI = 2.25; Miller & Thode 2007). To understand the sensitivity of our results to variation in this threshold, we refit the generalized linear models described in the main text across a sequence of threshold values from 2 to 2.5. As in section 4.3, we were unable to estimate robust standard errors across this range using spatial block bootstrapping. Instead, we report changes in the mean coefficient estimates (Fig. S8 and S10).

Variation in the CBI threshold affected the mean estimates of some model coefficients, but not enough to alter the main takeaways of our analysis. The effects which changed the most were: the ladder fuels index, which increased under higher thresholds; incoming severity, which decreased; and clustering, which increased (Fig. S8 and S10). The only key coefficient which changed sign, from negative to positive, was the main effect of average stem height. However, this was only true in the neighborhood-scale model (Fig. S8). The effect was relatively unchanged in the stand-scale model on which the analysis in the main text is focused (Fig. S10). Moreover, the interaction between stem height and hot-dry-windy index remained positive in both models, indicating that large trees increase high-severity fire occurrence in extreme weather conditions (Fig. S8 and S10). In sum, these changes do not alter the main takeaways of the study: the forest structure attributes more common on private industrial forests were those more associated with high-severity fire, and the effects of these attributes were amplified in extreme weather conditions.

It is not surprising that variation in the CBI threshold used to distinguish high-severity fire effects resulted in larger shifts in model coefficients than variation in subsampling intensity. The error in quantifying CBI increases substantially at high values, even after employing a bias correction (Miller & Thode 2007; Parks *et al.* 2019). Moreover, a CBI value of 2.25 correlates with live basal area mortality of approximately 95%, so higher thresholds attempt to discriminate among increasingly extreme levels of overstory mortality. From an ecological standpoint, it makes sense that the influence of forest structure, weather, and topography would differ when comparing fire effects above 95% mortality versus, for example, above 98%. However, because bias increases at high CBI values, it is difficult to attribute the observed changes in model coefficients to ecological or biophysical drivers rather than measurement error. Considering this,

the relatively small changes in coefficient estimates are further evidence that the results of this study are robust.

4.5 Multicollinearity among forest structure variables.

Many of the forest structure metrics we analyze in this manuscript are highly correlated with one another (Fig. S11 and S12). For example, areas with a high stem density tend to be more spatially homogenous and contain smaller average canopy gaps, because tree stems are ultimately discrete objects. Collinearity among predictors can bias the rankings of predictor importance in random forest models, such as those quantified in main text Figure 2. Despite the potential for bias, we retained all forest structure variables in the primary analysis because we were a priori interested in the impact of each variable on fire severity. To evaluate whether the ranking of predictors presented in the main text was robust to collinearity, we conducted a sensitivity analysis in which we removed highly correlated model features and compared the resulting importance rankings.

We performed this sensitivity analysis separately at the neighborhood and stand scales, removing features so that none of the remaining features had pairwise correlations greater than 0.5 (see Fig. S11 and S12). For the neighborhood-scale model, we removed mean gap area, spatial homogeneity, mean stem height, and heat load. For the stand scale model, we removed only spatial homogeneity and heat load. The resulting predictor importance rankings were nearly identical to those in the primary models, the absence of removed predictors notwithstanding (Fig. S13). The only changes in rank were: 1) in the neighborhood scale model, fuel moisture and fire ID were flipped, and 2) in the stand scale model, mean gap area, climatic water deficit, and slope were shuffled. However, these predictors had very similar Shapley values in both the full and reduced models, meaning the shifts could simply result from random fluctuations in the subset of data points selected for each Shapley feature importance calculation.

Fig. S1.
Forest structural characteristics at the neighborhood scale.

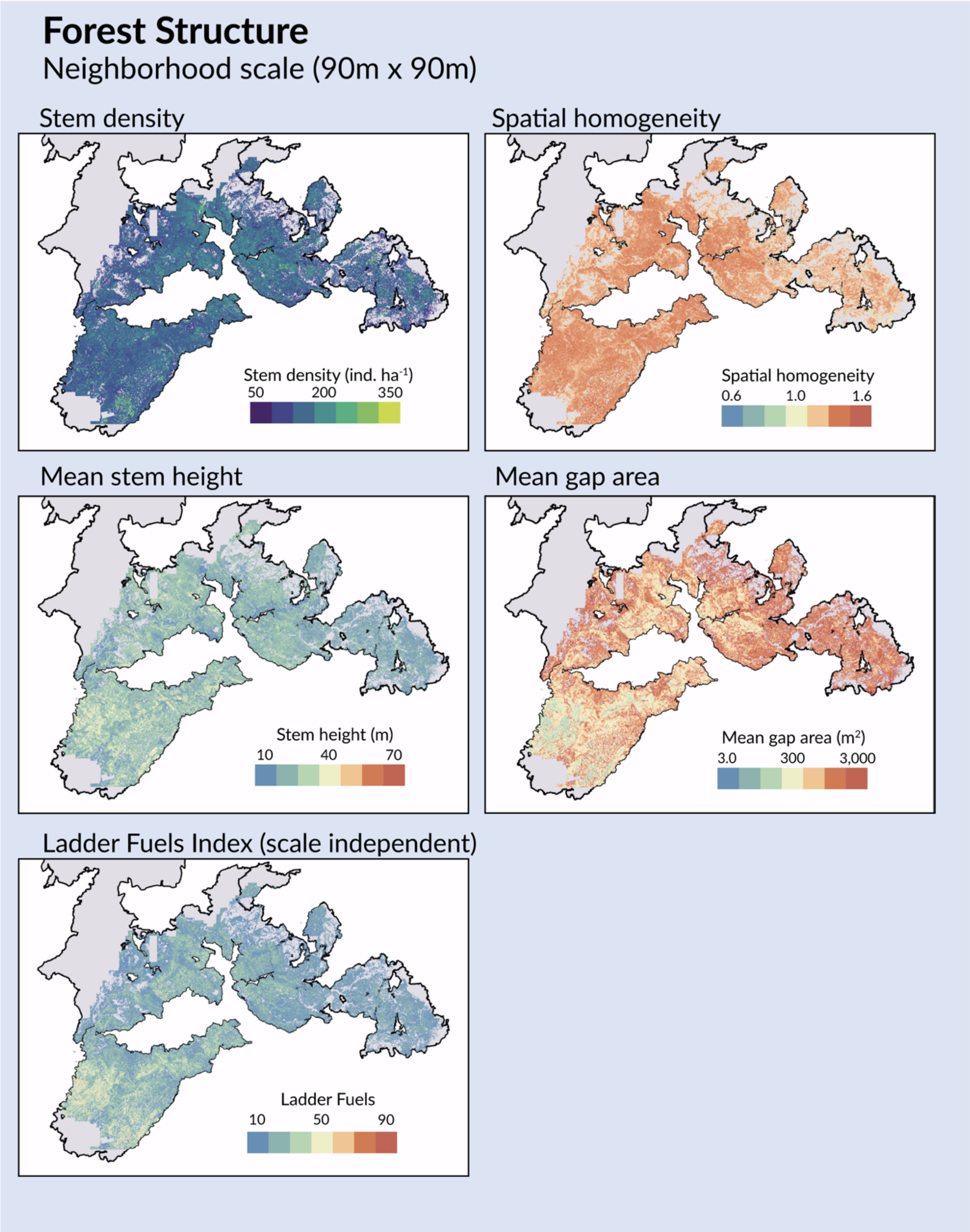

Fig. S2.
Forest structural characteristics at the stand scale.

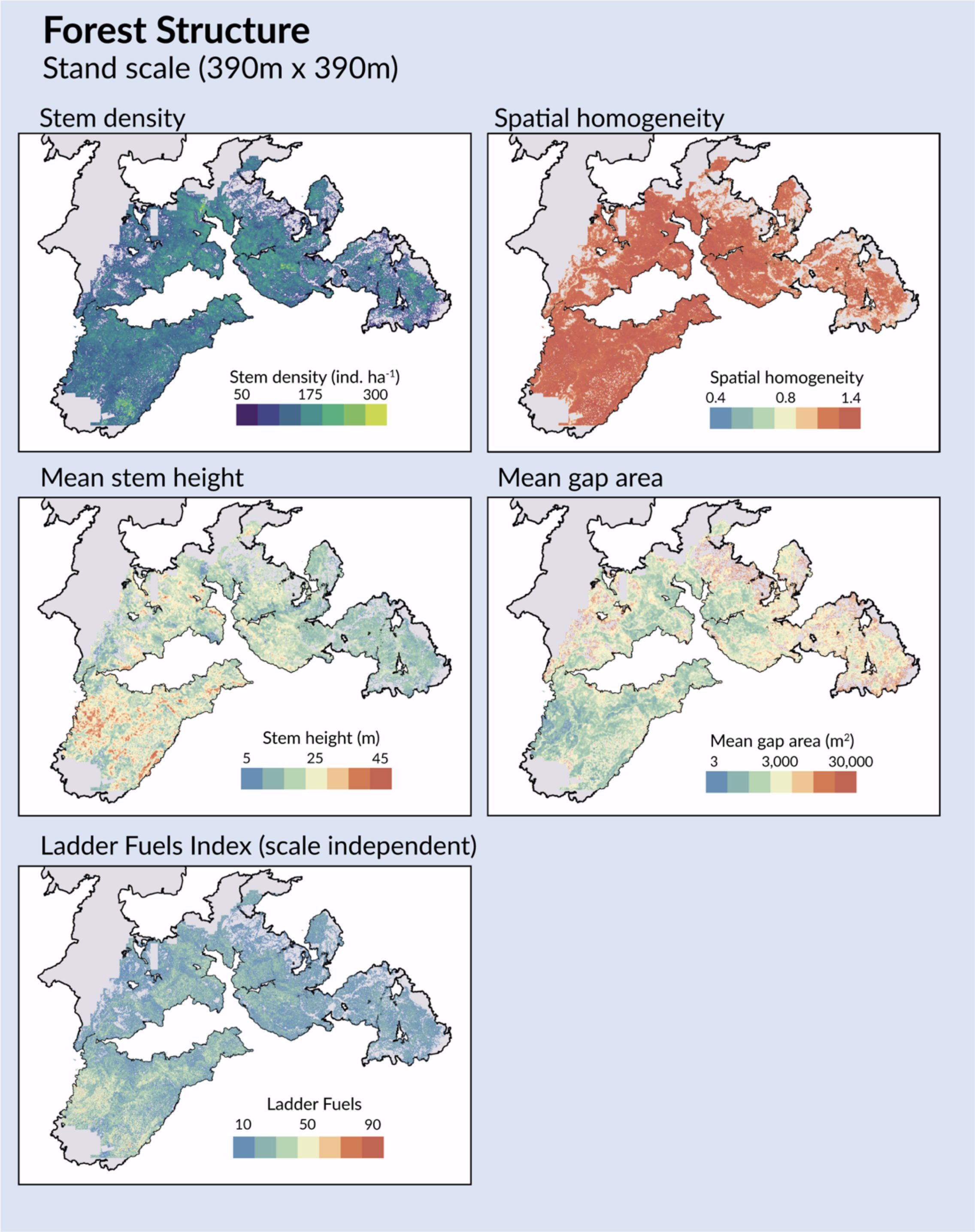

Fig. S3.
Weather, climate, and topographic variables.

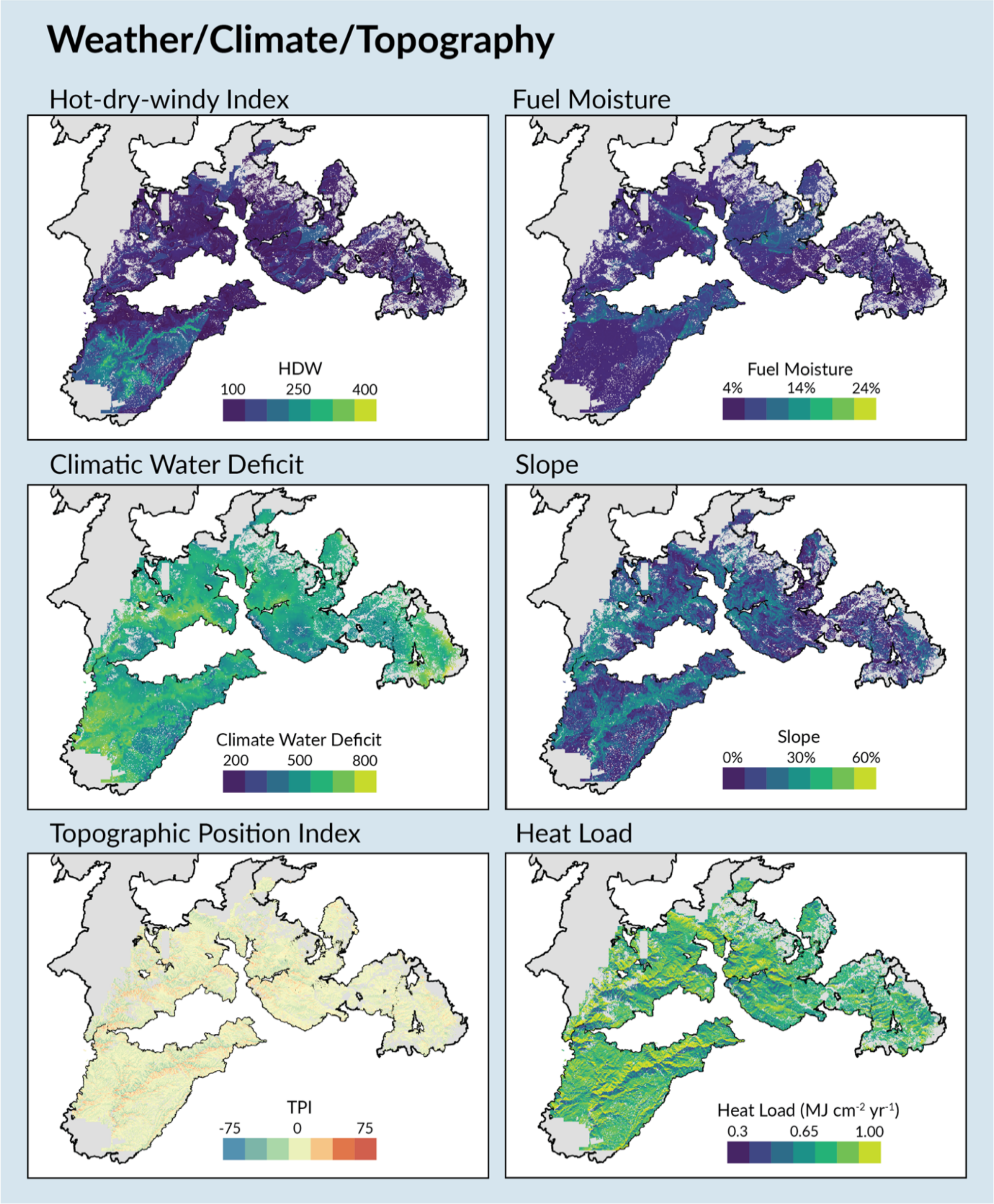

Fig. S4.
Fire behavior characteristics.

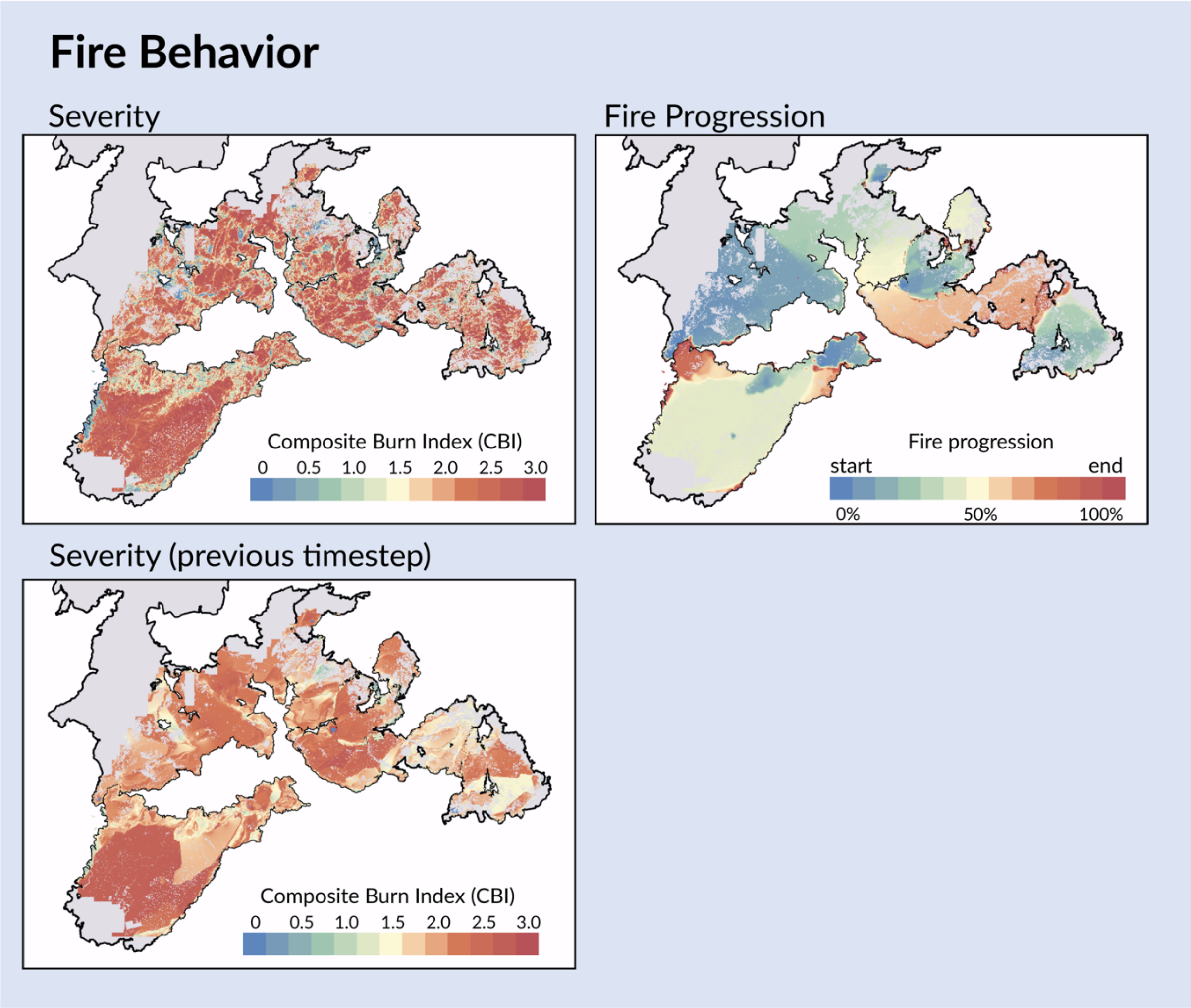

Fig. S5.
Location of remote automated weather stations (RAWS) used to calculate weather metrics.

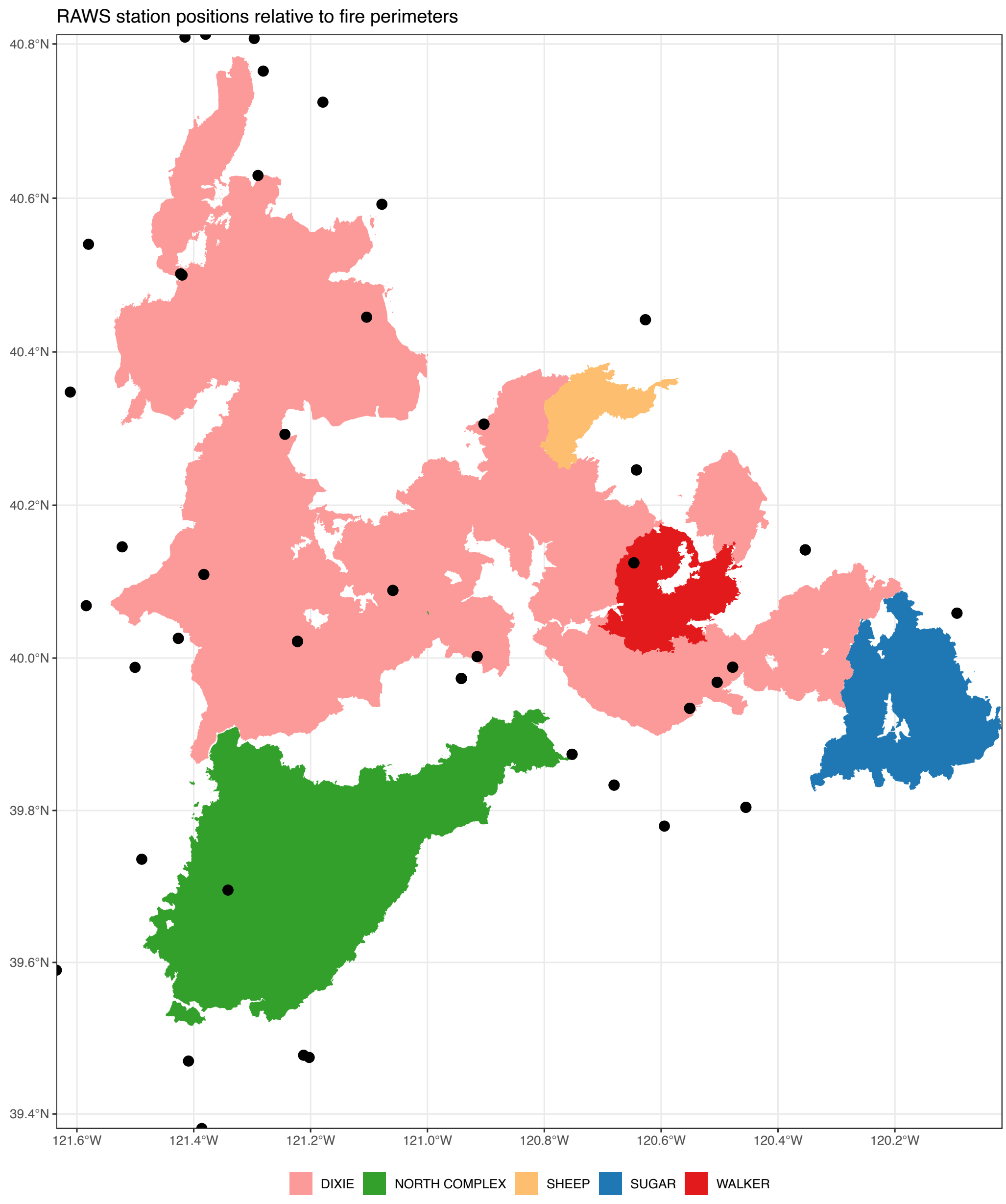

Fig. S6.
Estimated effects of forest structure, weather, and topography on the probability of a 30m by 30m pixel burning at high-severity for the neighborhood scale (90m by 90m) model.

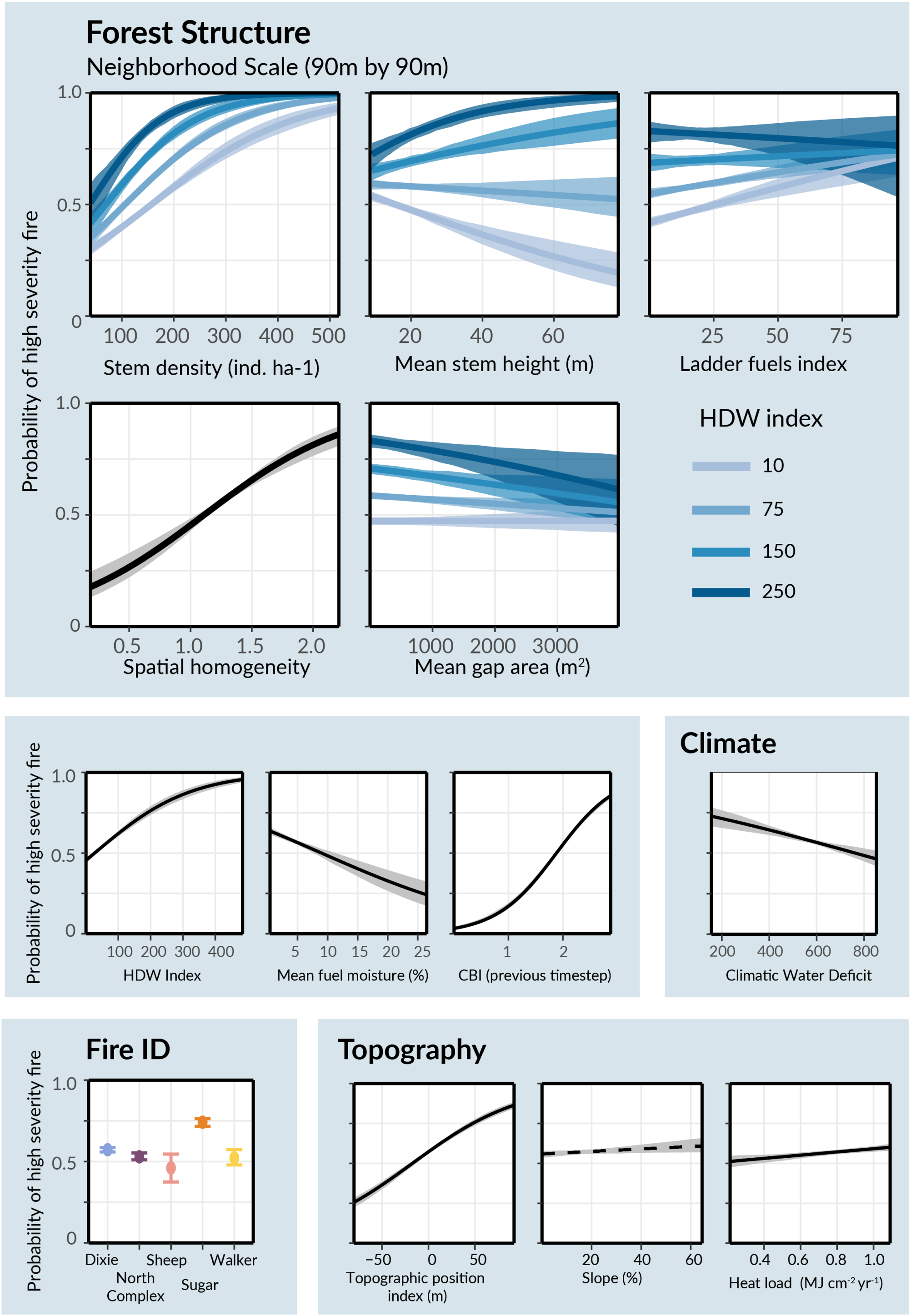

Figure S7.

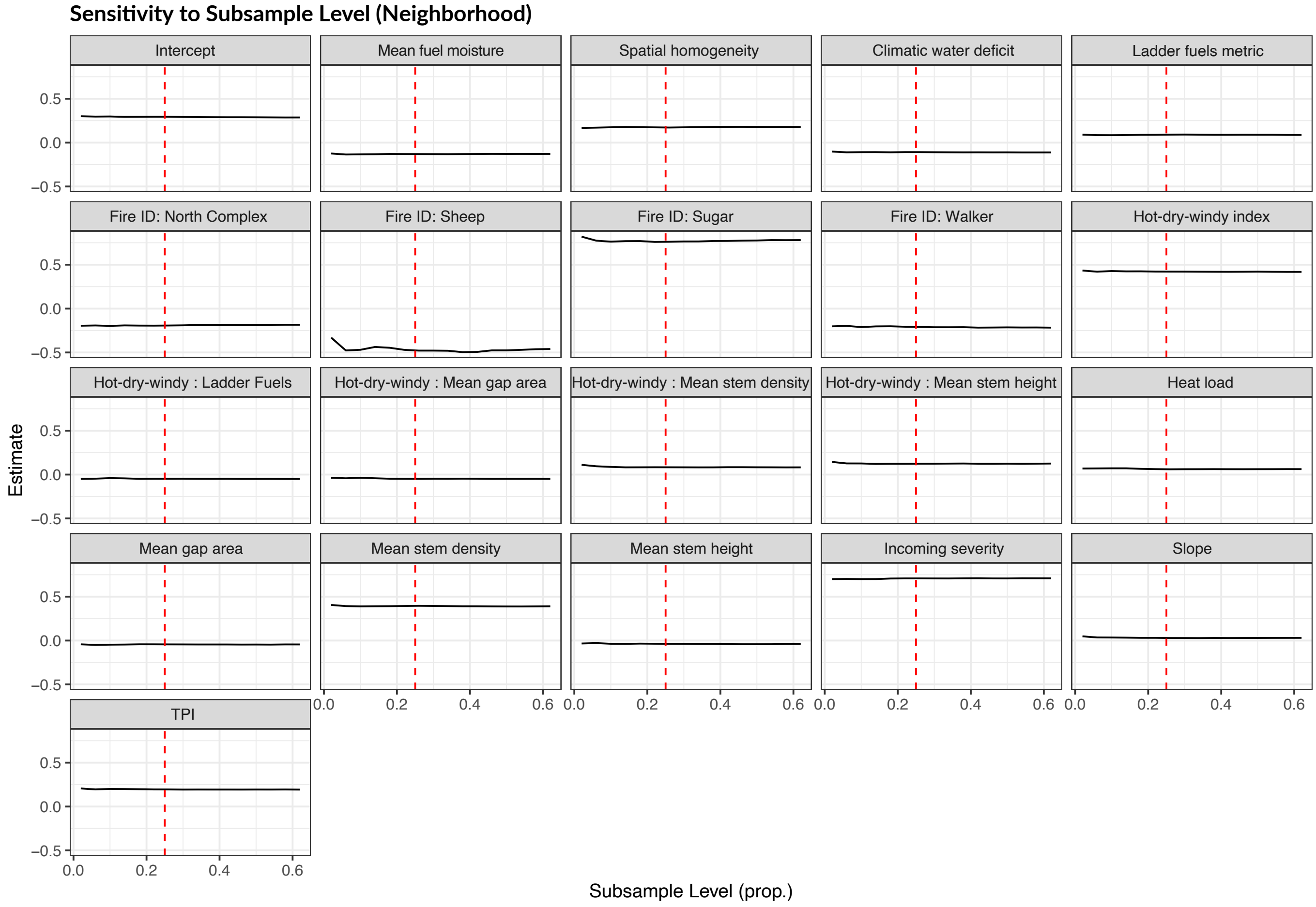
Sensitivity to Subsample Level (Neighborhood)
Intercept
Mean fuel moisture
Spatial homogeneity
Climatic water deficit
Ladder fuels metric
Fire ID: North Complex
Fire ID: Sheep
Fire ID: Sugar
Fire ID: Walker
Hot-dry-windy index
Hot-dry-windy : Ladder Fuels
Hot-dry-windy : Mean gap area
Hot-dry-windy : Mean stem density
Hot-dry-windy : Mean stem height
Heat load
Mean gap area
Mean stem density
Mean stem height
Incoming severity
Slope
TPI
Estimate
Subsample Level (prop.)
0.5
0.0
−0.5
0.0
0.2
0.4
0.6

Figure S8.

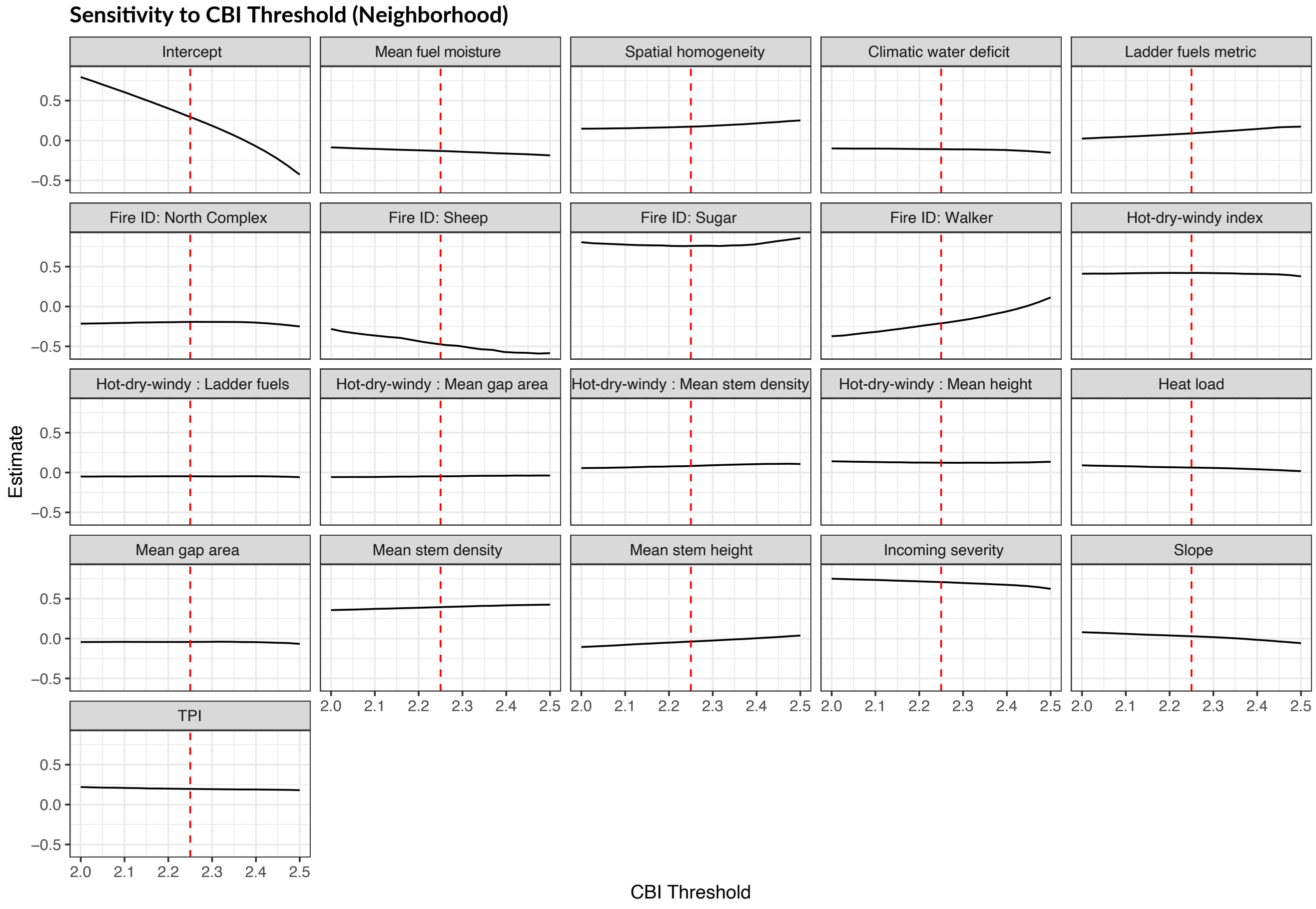
Sensitivity to CBI Threshold (Neighborhood)
Intercept
Mean fuel moisture
Spatial homogeneity
Climatic water deficit
Ladder fuels metric
Fire ID: North Complex
Fire ID: Sheep
Fire ID: Sugar
Fire ID: Walker
Hot-dry-windy index
Hot-dry-windy : Ladder fuels
Hot-dry-windy : Mean gap area
Hot-dry-windy : Mean stem density
Hot-dry-windy : Mean height
Heat load
Mean gap area
Mean stem density
Mean stem height
Incoming severity
Slope
TPI
Estimate
0.5
0.0
−0.5
2.0
2.1
2.2
2.3
2.4
2.5
CBI Threshold

Figure S9.

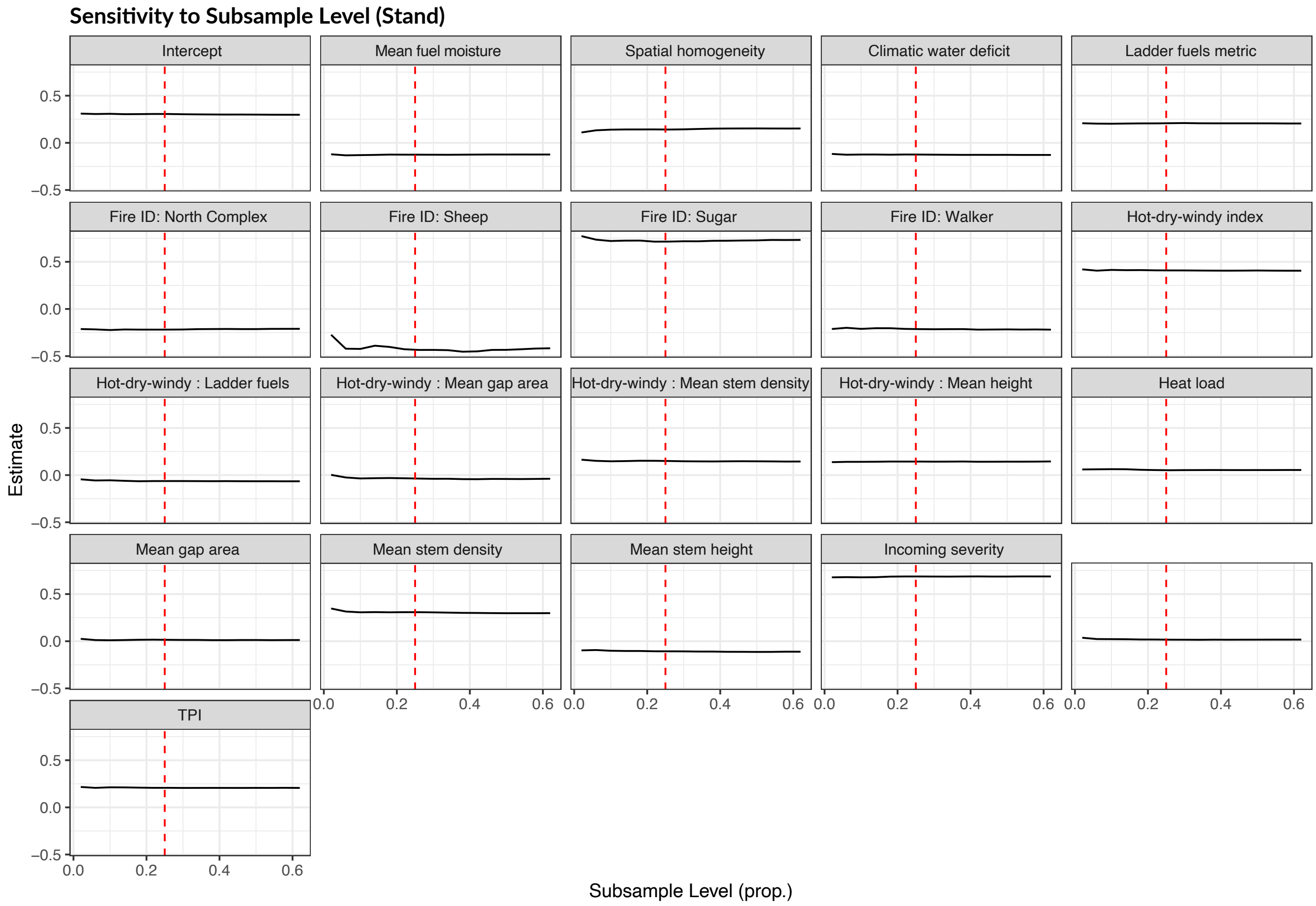

Sensitivity to Subsample Level (Stand)
Intercept
Mean fuel moisture
Spatial homogeneity
Climatic water deficit
Ladder fuels metric
Fire ID: North Complex
Fire ID: Sheep
Fire ID: Sugar
Fire ID: Walker
Hot-dry-windy index
Hot-dry-windy : Ladder fuels
Hot-dry-windy : Mean gap area
Hot-dry-windy : Mean stem density
Hot-dry-windy : Mean height
Heat load
Mean gap area
Mean stem density
Mean stem height
Incoming severity
TPI
Estimate
Subsample Level (prop.)

Figure S10.

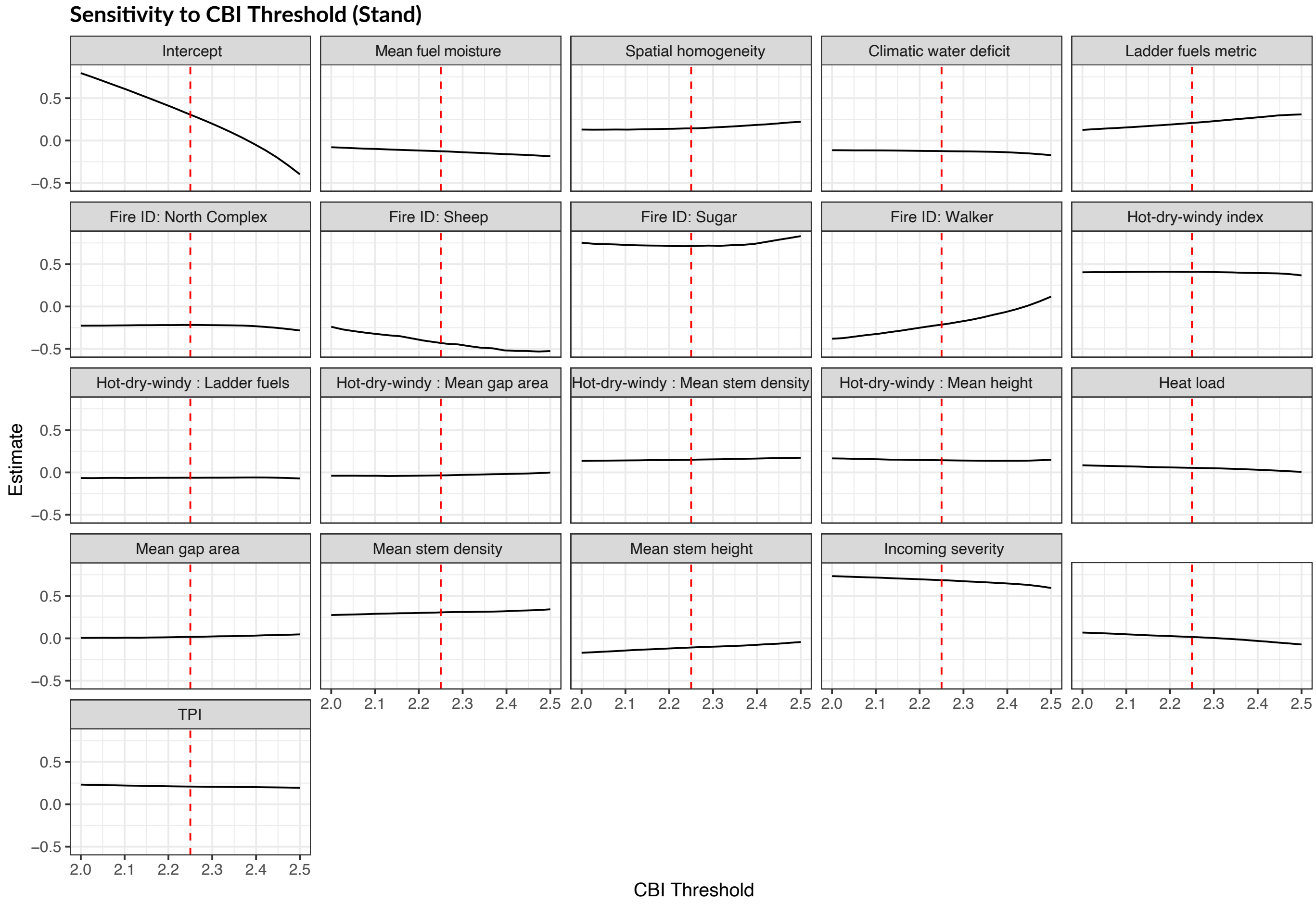

Sensitivity to CBI Threshold (Stand)
Intercept
Mean fuel moisture
Spatial homogeneity
Climatic water deficit
Ladder fuels metric
Fire ID: North Complex
Fire ID: Sheep
Fire ID: Sugar
Fire ID: Walker
Hot-dry-windy index
Hot-dry-windy : Ladder fuels
Hot-dry-windy : Mean gap area
Hot-dry-windy : Mean stem density
Hot-dry-windy : Mean height
Heat load
Mean gap area
Mean stem density
Mean stem height
Incoming severity
TPI
Estimate
0.5
0.0
−0.5
2.0
2.1
2.2
2.3
2.4
2.5
CBI Threshold

Figure S11.

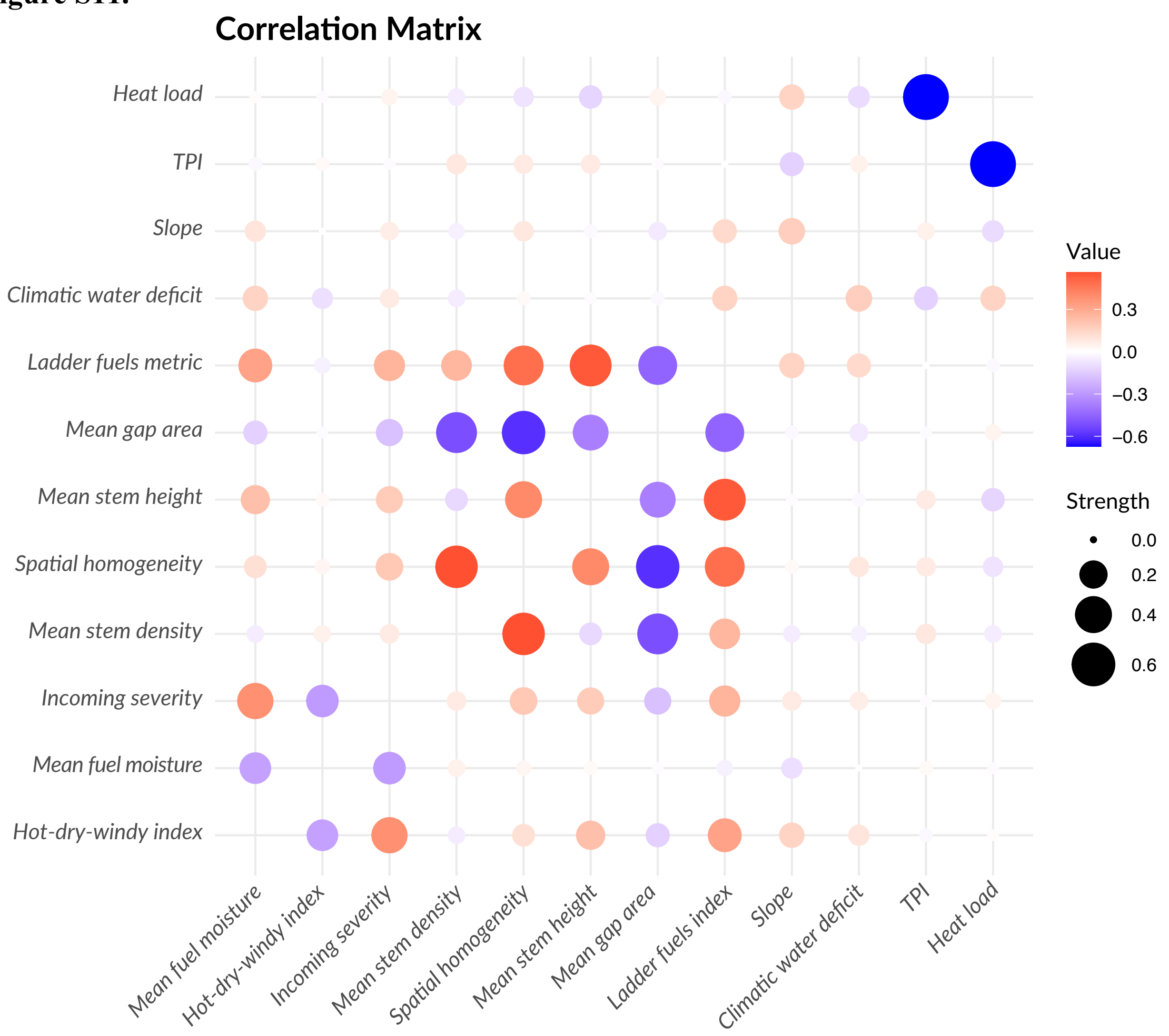

Correlation Matrix
Heat load
TPI
Slope
Climatic water deficit
Ladder fuels metric
Mean gap area
Mean stem height
Spatial homogeneity
Mean stem density
Incoming severity
Mean fuel moisture
Hot-dry-windy index
Mean fuel moisture
Hot-dry-windy index
Incoming severity
Mean stem density
Spatial homogeneity
Mean stem height
Mean gap area
Ladder fuels index
Slope
Climatic water deficit
TPI
Heat load
Value
0.3
0.0
−0.3
−0.6
Strength
0.0
0.2
0.4
0.6

Figure S12.

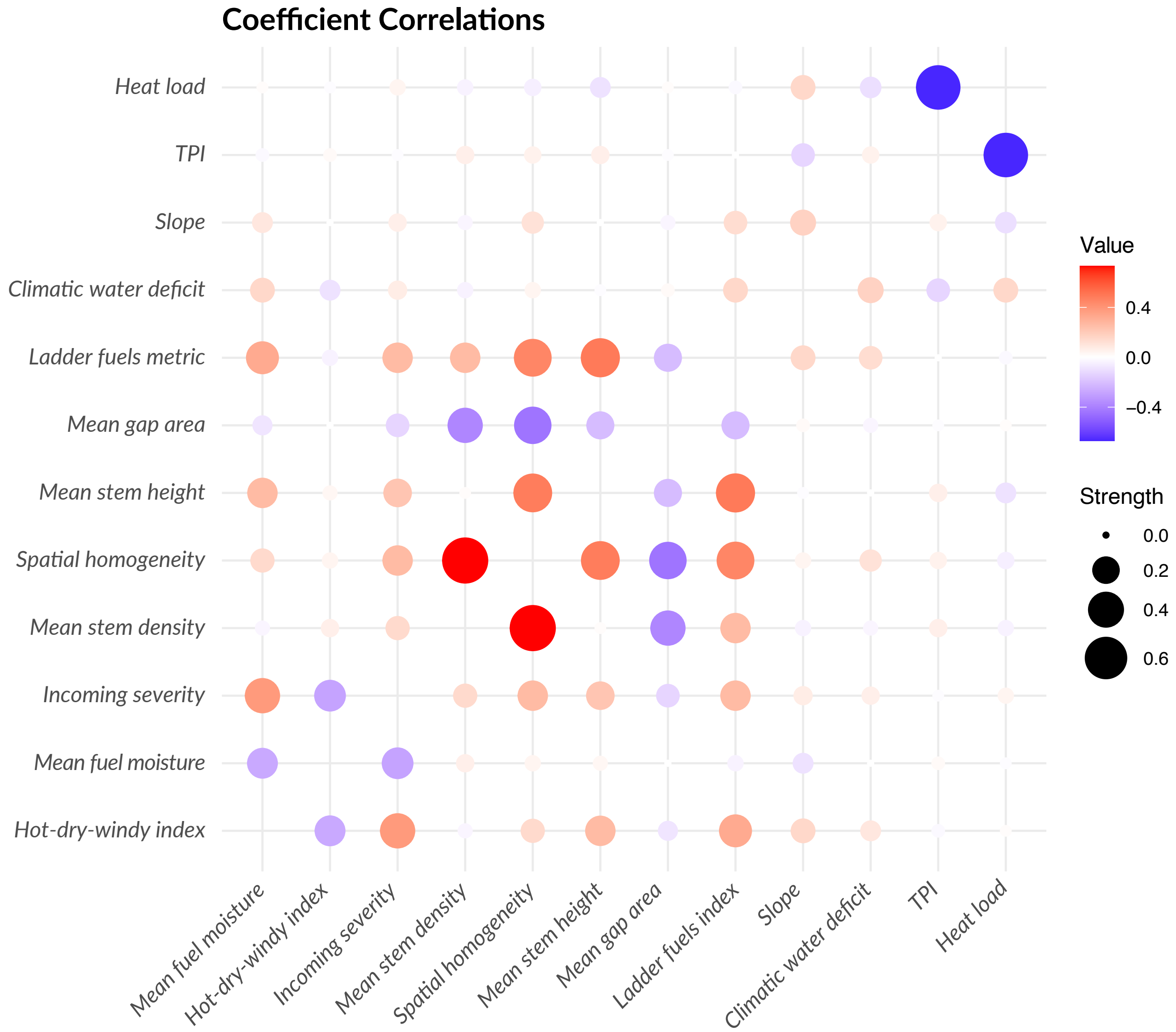
Coefficient Correlations
Heat load
TPI
Slope
Climatic water deficit
Ladder fuels metric
Mean gap area
Mean stem height
Spatial homogeneity
Mean stem density
Incoming severity
Mean fuel moisture
Hot-dry-windy index
Mean fuel moisture
Hot-dry-windy index
Incoming severity
Mean stem density
Spatial homogeneity
Mean stem height
Mean gap area
Ladder fuels index
Slope
Climatic water deficit
TPI
Heat load
Value
0.4
0.0
−0.4
Strength
0.0
0.2
0.4
0.6

Figure S13. Shapley importance rankings after removing variables with correlations greater than 0.5.

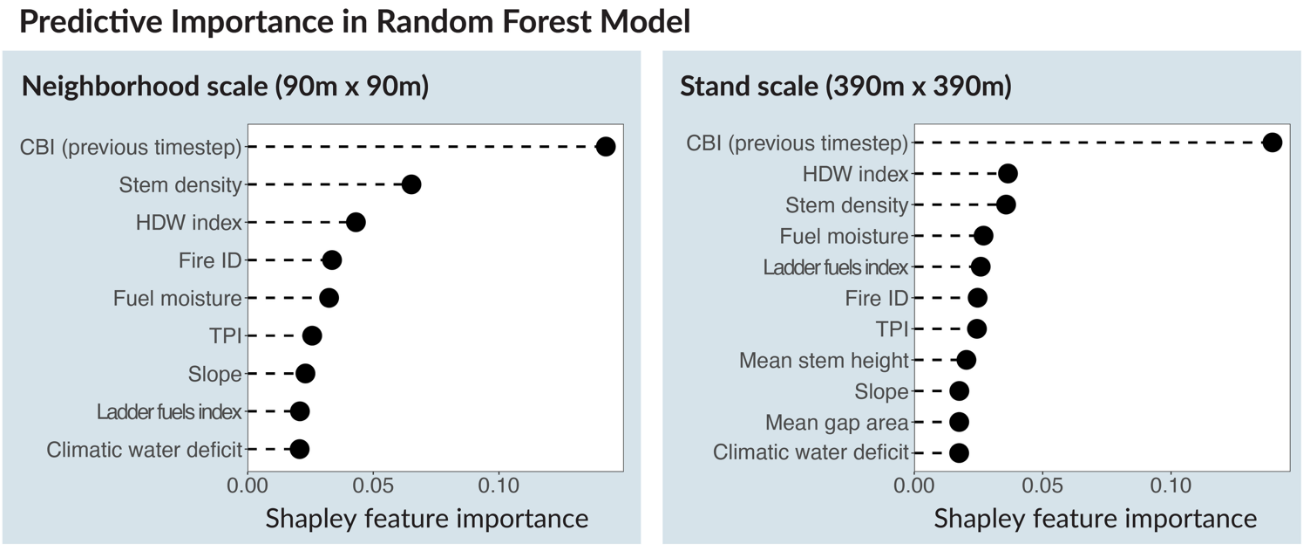

Table S1.

Summary of wildfires included in the study. Statistics represent summaries for areas of the fire within the study area (area coincident with 2018 LiDAR flight/Plumas County, CA). All statistics include only areas that were forested before each fire.

Fire Name	Year	Forested area (hectares)	% high severity	% public	% private industrial
Dixie	2021	154,907	50.4	86.1	8.5
North Complex	2020	105,195	63.1	70.6	21.9
Sugar	2021	25,683	54.4	88.2	0.0
Walker	2019	20,450	44.1	98.7	0.0
Sheep	2020	2,557	48.8	82.3	16.6

Table S2.

Summary of estimated effects and confidence intervals for the binomial GLM relating ownership to high-severity fire incidence.

Variable	Estimate	95% C.I. (lower)	95% C.I. (upper)
Intercept	0.26	0.20	0.31
Ownership: Other	-0.39	-0.51	-0.28
Ownership: Private Industrial	0.37	0.26	0.48
Hot-dry-windy index	0.39	0.33	0.44
Fuel moisture	-0.12	-0.15	-0.08
Climatic water deficit	-0.057	-0.09	-0.02
Slope	0.025	-0.01	0.06
TPI	0.22	0.20	0.24
Heat load	0.036	0.004	0.07
CBI (prev. timestep)	0.73	0.69	0.79
Fire ID: North Complex	-0.09	-0.18	0.03
Fire ID: Sheep	-0.53	-0.89	-0.16
Fire ID: Sugar	0.59	0.45	0.73
Fire ID: Walker	-0.18	-0.33	-0.01

Table S3.

Confusion matrix for neighborhood scale (90m x 90m) random forest model.

	Ground Truth	
**Predicted**	High	Low-moderate
High severity	288,126	73,597
Not high severity	81,456	220,698

Table S4.

Confusion matrix for stand scale (390m x 390m) random forest model.

	**Ground Truth**	
**Predicted**	High	Low-moderate
High severity	287,806	67,760
Not high severity	81,776	226,535

Table S5.

Summary of estimated effects and confidence intervals for the neighborhood scale (90m by 90m) binomial GLM relating forest structure, weather and topography to high-severity fire incidence.

Variable	Estimate	95% C.I. (lower)	95% C.I. (upper)
Intercept	0.29	0.23	0.34
**Weather/Fire Behavior**			
Hot-dry-windy index	0.42	0.37	0.49
Fuel moisture	-0.13	-0.17	-0.1
CBI (prev. timestep)	0.70	0.65	0.75
**Forest Structure**			
Mean stem density	0.40	0.35	0.46
Spatial homogeneity	0.17	0.13	0.21
Mean stem height	-0.03	-0.07	0.007
Mean area	-0.04	-0.07	-0.009
Ladder fuels index	0.09	0.05	0.13
**Climate/Topography**			
Climatic water deficit	-0.11	-0.15	-0.06
Slope	0.03	-0.00009	0.06
TPI	0.19	0.17	0.21
Heat load	0.06	0.03	0.09
**Interactions with Hot-Dry-Windy Index**			
Mean stem density : Hot-dry-windy index	0.09	0.04	0.14
Mean stem height : Hot-dry-windy index	0.13	0.08	0.17
Mean gap area : Hot-dry-windy index	-0.05	-0.08	-0.01
Ladder fuels index : Hot-dry-windy index	-0.05	-0.09	-0.003
**Fire ID**			
Fire ID: North Complex	-0.18	-0.28	-0.07
Fire ID: Sheep	-0.45	-0.10	-0.12
Fire ID: Sugar	0.76	0.61	0.89
Fire ID: Walker	-0.20	-0.38	-0.0003

Table S6.

Summary of estimated effects and confidence intervals for the stand scale (390m by 390m) binomial GLM relating forest structure, weather and topography to high-severity fire incidence.

Variable	Estimate	95% C.I. (lower)	95% C.I. (upper)
Intercept	0.31	0.24	0.36
**Weather/Fire Behavior**			
Hot-dry-windy index	0.41	0.35	0.46
Fuel moisture	-0.13	-0.17	-0.09
CBI (prev. timestep)	0.68	0.63	0.74
**Forest Structure**			
Mean stem density	0.32	0.25	0.40
Spatial homogeneity	0.13	0.06	0.20
Mean stem height	-0.10	-0.16	-0.04
Mean area	0.017	-0.015	0.045
Ladder fuels index	0.21	0.17	0.24
**Climate/Topography**			
Climatic water deficit	-0.12	-0.16	-0.07
Slope	0.019	-0.017	0.06
TPI	0.21	0.19	0.22
Heat load	0.054	0.02	0.084
**Interactions with Hot-Dry-Windy Index**			
Mean stem density : Hot-dry-windy index	0.15	0.098	0.21
Mean stem height : Hot-dry-windy index	0.15	0.11	0.20
Mean gap area : Hot-dry-windy index	-0.04	-0.08	0.004
Ladder fuels index : Hot-dry-windy index	-0.06	-0.10	-0.02
**Fire ID**			
Fire ID: North Complex	-0.21	-0.31	-0.08
Fire ID: Sheep	-0.41	-0.76	-0.08
Fire ID: Sugar	0.71	0.59	0.84
Fire ID: Walker	-0.21	-0.37	-0.05

Table S7.
Confusion matrix for stand scale (380m x 380m) random forest model, when trained on a subset of fires (Dixie, North Complex, Sugar), and tested on the remaining ones (Sheep and Walker). The AUC was 0.7725.

	Ground Truth	
**Predicted**	High	Low-moderate
High severity	87,720	50,677
Not high severity	13,633	60,520

Table S8.

Summary of estimated effects and confidence intervals for the binomial GLM relating ownership to high-severity fire incidence ***as classified using dNBR instead of CBI***.

Variable	Estimate	95% C.I. (lower)	95% C.I. (upper)
Intercept	0.44	0.40	0.51
Ownership: Other	-0.32	-0.44	-0.21
Ownership: Private Industrial	0.38	0.27	0.47
Hot-dry-windy index	0.37	0.31	0.43
Fuel moisture	-0.03	-0.07	0.00
Climatic water deficit	-0.04	-0.08	0.00
Slope	0.07	0.04	0.09
TPI	0.21	0.19	0.24
Heat load	0.05	0.02	0.08
Incoming severity	0.78	0.74	0.82
Fire ID: North Complex	0.04	-0.04	0.14
Fire ID: Sheep	-0.77	-1.09	-0.46
Fire ID: Sugar	1.47	1.34	1.66
Fire ID: Walker	-0.63	-0.79	-0.48

Table S9.

Summary of estimated effects and confidence intervals for the neighborhood scale (90m by 90m) binomial GLM relating forest structure, weather and topography to high-severity fire incidence ***as classified using dNBR instead of CBI***.

Variable	Estimate	95% C.I. (lower)	95% C.I. (upper)
Intercept	0.50	0.45	0.55
**Weather/Fire Behavior**			
Hot-dry-windy index	0.43	0.38	0.50
Fuel moisture	-0.05	-0.08	-0.01
Incoming severity	0.75	0.70	0.81
**Forest Structure**			
Mean stem density	0.42	0.38	0.47
Spatial homogeneity	0.15	0.12	0.19
Mean stem height	-0.03	-0.07	0.02
Mean area	-0.05	-0.08	-0.02
Ladder fuels index	0.06	0.03	0.10
**Climate/Topography**			
Climatic water deficit	-0.09	-0.13	-0.05
Slope	0.08	0.05	0.11
TPI	0.18	0.16	0.20
Heat load	0.06	0.03	0.09
**Interactions with Hot-Dry-Windy Index**			
Mean stem density : Hot-dry-windy index	0.10	0.05	0.15
Mean stem height : Hot-dry-windy index	0.15	0.10	0.19
Mean gap area : Hot-dry-windy index	-0.04	-0.07	0.01
Ladder fuels index : Hot-dry-windy index	-0.09	-0.13	-0.05
**Fire ID**			
Fire ID: North Complex	-0.03	-0.11	0.06
Fire ID: Sheep	-0.74	-1.08	-0.35
Fire ID: Sugar	1.71	1.58	1.85
Fire ID: Walker	-0.69	-0.86	-0.50

Table S10.

Summary of estimated effects and confidence intervals for the stand scale (390m by 390m) binomial GLM relating forest structure, weather and topography to high-severity fire incidence ***as classified using dNBR instead of CBI***.

Variable	Estimate	95% C.I. (lower)	95% C.I. (upper)
Intercept	0.51	0.47	0.58
**Weather/Fire Behavior**			
Hot-dry-windy index	0.41	0.36	0.47
Fuel moisture	-0.05	-0.07	-0.01
Incoming severity	0.73	0.67	0.79
**Forest Structure**			
Mean stem density	0.35	0.28	0.42
Spatial homogeneity	0.10	0.04	0.17
Mean stem height	-0.08	-0.13	-0.02
Mean area	0.02	0.00	0.04
Ladder fuels index	0.18	0.15	0.22
**Climate/Topography**			
Climatic water deficit	-0.09	-0.13	-0.06
Slope	0.07	0.04	0.10
TPI	0.20	0.18	0.23
Heat load	0.06	0.03	0.09
**Interactions with Hot-Dry-Windy Index**			
Mean stem density : Hot-dry-windy index	0.17	0.12	0.22
Mean stem height : Hot-dry-windy index	0.16	0.11	0.20
Mean gap area : Hot-dry-windy index	0.00	-0.04	0.02
Ladder fuels index : Hot-dry-windy index	-0.10	-0.14	-0.06
**Fire ID**			
Fire ID: North Complex	-0.06	-0.16	0.02
Fire ID: Sheep	-0.79	-1.10	-0.42
Fire ID: Sugar	1.62	1.45	1.76
Fire ID: Walker	-0.67	-0.81	-0.53

Table S11.

Summary of estimated effects and confidence intervals for the binomial GLM relating ownership to high-severity fire incidence ***after removing climatic water deficit as a covariate***.

Variable	Estimate	95% C.I. (lower)	95% C.I. (upper)
Intercept	0.26	0.21	0.31
Ownership: Other	-0.42	-0.53	-0.32
Ownership: Private Industrial	0.38	0.27	0.49
Hot-dry-windy index	0.37	0.31	0.42
Fuel moisture	-0.11	-0.15	-0.08
Slope	0.01	-0.03	0.05
TPI	0.23	0.21	0.25
Heat load	0.03	0.00	0.06
CBI (prev. timestep)	0.73	0.68	0.78
Fire ID: North Complex	-0.09	-0.22	0.02
Fire ID: Sheep	-0.54	-0.82	-0.14
Fire ID: Sugar	0.55	0.39	0.66
Fire ID: Walker	-0.20	-0.37	-0.04

Table S12.

Summary of estimated effects and confidence intervals for the neighborhood scale (90m by 90m) binomial GLM relating forest structure, weather and topography to high-severity fire incidence ***after removing climatic water deficit as a covariate***.

Variable	Estimate	95% C.I. (lower)	95% C.I. (upper)
Intercept	0.30	0.26	0.37
**Weather/Fire Behavior**			
Hot-dry-windy index	0.41	0.36	0.47
Fuel moisture	-0.13	-0.17	-0.08
CBI (prev. timestep)	0.71	0.65	0.77
**Forest Structure**			
Mean stem density	0.41	0.37	0.47
Spatial homogeneity	0.16	0.13	0.19
Mean stem height	-0.02	-0.07	0.02
Mean area	-0.04	-0.06	-0.01
Ladder fuels index	0.07	0.03	0.11
**Climate/Topography**			
Slope	0.01	-0.02	0.05
TPI	0.20	0.18	0.23
Heat load	0.05	0.02	0.08
**Interactions with Hot-Dry-Windy Index**			
Mean stem density : Hot-dry-windy index	0.11	0.06	0.16
Mean stem height : Hot-dry-windy index	0.14	0.09	0.18
Mean gap area : Hot-dry-windy index	-0.04	-0.07	-0.01
Ladder fuels index : Hot-dry-windy index	-0.06	-0.10	-0.02
**Fire ID**			
Fire ID: North Complex	-0.16	-0.27	-0.05
Fire ID: Sheep	-0.41	-0.81	-0.01
Fire ID: Sugar	0.66	0.53	0.81
Fire ID: Walker	-0.21	-0.39	-0.04

Table S13.

Summary of estimated effects and confidence intervals for the stand scale (390m by 390m) binomial GLM relating forest structure, weather and topography to high-severity fire incidence ***after removing climatic water deficit as a covariate.***

Variable	Estimate	95% C.I. (lower)	95% C.I. (upper)
Intercept	0.32	0.26	0.37
**Weather/Fire Behavior**			
Hot-dry-windy index	0.40	0.36	0.45
Fuel moisture	-0.12	-0.16	-0.09
CBI (prev. timestep)	0.68	0.63	0.74
**Forest Structure**			
Mean stem density	0.33	0.26	0.40
Spatial homogeneity	0.12	0.03	0.20
Mean stem height	-0.10	-0.15	-0.04
Mean area	0.02	-0.01	0.04
Ladder fuels index	0.19	0.16	0.22
**Climate/Topography**			
Slope	0.00	-0.04	0.03
TPI	0.22	0.20	0.24
Heat load	0.03	0.00	0.07
**Interactions with Hot-Dry-Windy Index**			
Mean stem density : Hot-dry-windy index	0.16	0.11	0.21
Mean stem height : Hot-dry-windy index	0.14	0.10	0.18
Mean gap area : Hot-dry-windy index	-0.03	-0.08	0.01
Ladder fuels index : Hot-dry-windy index	-0.07	-0.11	-0.03
**Fire ID**			
Fire ID: North Complex	-0.21	-0.32	-0.11
Fire ID: Sheep	-0.42	-0.80	0.02
Fire ID: Sugar	0.62	0.50	0.73
Fire ID: Walker	-0.21	-0.35	-0.06
